# Unsupervised discovery of solid-state lithium ion conductors

**DOI:** 10.1038/s41467-019-13214-1

**Published:** 2019-11-20

**Authors:** Ying Zhang, Xingfeng He, Zhiqian Chen, Qiang Bai, Adelaide M. Nolan, Charles A. Roberts, Debasish Banerjee, Tomoya Matsunaga, Yifei Mo, Chen Ling

**Affiliations:** 1 Toyota Research Institute of North America, Ann Arbor, MI 48105 USA; 2 Department of Materials Science and Engineering, University of Maryland, College Park, MD 20742 USA; 3 Department of Computer Science, Virginia Tech, 7054 Haycock Road, Falls Church, VA 2043 USA; 4 Maryland Energy Innovation Institute, University of Maryland, College Park, MD 20742 USA

**Keywords:** Solid-state chemistry, Materials for energy and catalysis, Theory and computation

## Abstract

Although machine learning has gained great interest in the discovery of functional materials, the advancement of reliable models is impeded by the scarcity of available materials property data. Here we propose and demonstrate a distinctive approach for materials discovery using unsupervised learning, which does not require labeled data and thus alleviates the data scarcity challenge. Using solid-state Li-ion conductors as a model problem, unsupervised materials discovery utilizes a limited quantity of conductivity data to prioritize a candidate list from a wide range of Li-containing materials for further accurate screening. Our unsupervised learning scheme discovers 16 new fast Li-conductors with conductivities of 10^−4^–10^−1^ S cm^−1^ predicted in ab initio molecular dynamics simulations. These compounds have structures and chemistries distinct to known systems, demonstrating the capability of unsupervised learning for discovering materials over a wide materials space with limited property data.

## Abstract (web summary)

Predictions of new solid-state Li-ion conductors are challenging due to the diverse chemistries and compositions involved. Here the authors combine unsupervised learning techniques and molecular dynamics simulations to discover new compounds with high Li-ion conductivity.

## Introduction

**Fig. 1:**
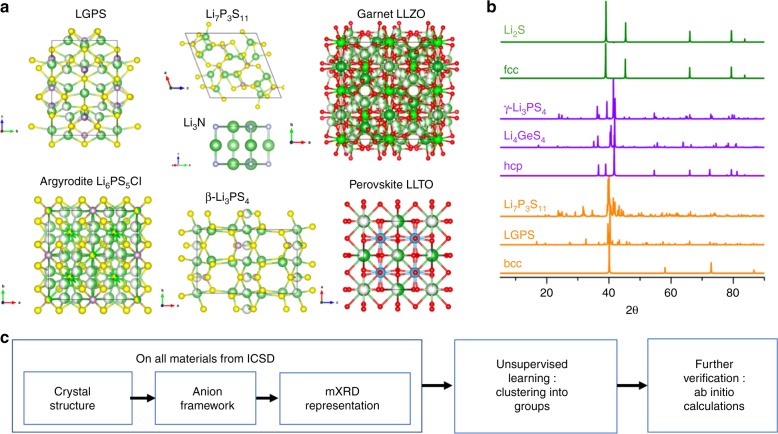
Schematics of the unsupervised discovery of solid-state Li-ion conductors. **a** Crystal structures of known SSLCs, showing a large diversity of structure and chemistry. **b** mXRD patterns of selected materials in comparison to those of ideal fcc (face centered cubic), hcp (hexagonal close packed), bcc (body centered cubic) lattices. **c** Workflow of an unsupervised learning guided discovery of SSLCs

The fast conduction of lithium (Li) ions in a solid is a phenomenon of significant scientific interest and technological importance. The room-temperature Li-ion conductivities (*σ*_RT_) in poorly conductive and fast conducting materials can differ by more than twenty orders of magnitude^1,2^. The high *σ*_RT_ in electrode and electrolyte materials are essential for high power/rate performance of batteries. In particular, replacing the flammable liquid electrolyte used in commercial Li-ion batteries with a fast Li-conducting solid electrolyte, to produce an all-solid-state battery, provides improved safety, excellent stability, and long cycling life^1,2^. Although there are several thousands of known lithium-containing compounds, fast Li^+^ conduction with *σ*_RT_ close to 10^−3^–10^−2^ S cm^−1^, comparable to the level in liquid electrolytes, is a rare property held by only a few solid-state Li-ion conductors (SSLCs), such as lithium thiophosphates (e.g., Li_7_P_3_S_11_^3^ and Li_10_GeP_2_S_12_^4^, LGPS), garnet (e.g., Li_7_Li_3_Zr_2_O_12_^5^, LLZO), NASICON (e.g., Li_1.3_Al_0.3_Ti_1.7_(PO_4_)_3_^6^, LATP), perovskite (e.g., Li_0.5_La_0.5_TiO_3_^7^, LLTO), Li_3_N^8^, and argyrodite^9^ (e.g., Li_6_PS_5_Cl) (Fig. 1a). Since these known SSLCs do not meet all desired attributes required for the commercialization of all-solid-state batteries^10^, there is significant interest in discovering new SSLC materials with high *σ*_RT_. The challenges in predicting new SSLCs are largely a result of the diverse chemistries and structures of SSLCs, and current computational predictions and laboratory syntheses are often performed on a limited number of candidates^1,2^. SSLCs have compositions ranging from oxides and sulfides to nitrides and mixed halides, and a diverse set of crystalline structures including perovskite, argyrodite, garnet, and NASICON, and newly discovered structures, such as LGPS and Li_7_P_3_S_11_. Over the past few years, first-principles computation has played an important role in the successful prediction of a number of novel SSLCs^11–15^. Recent studies have determined a number of key physical factors required for fast Li-ion diffusion, such as anion lattice packing^13^, lattice dynamics^16,17^, frustration of the mobile-ion sublattice^18,19^, and concerted ion migration^14^. So far, transforming a theory into a predictive model to explore a vast composition-structure space of many materials remains a significant challenge.

Machine learning (ML) has emerged as a technique for materials discovery thanks to its capability of recognizing complex patterns in data^20–28^ by representing materials with critical descriptors such as the combination of chemistry, composition, and crystal structure that yields desired materials properties. While significant research progress has been achieved by improving the materials descriptors over the years^29–37^, the applications of ML for materials discovery is in general plagued by two significant challenges. First, a ML model requires training on a sufficient amount of data to capture the correlation between a desired physical property and the features of materials. Unfortunately, only a few select materials exhibit the property of interest, as is often the case in materials discovery. In many cases, even the data for materials with poor properties is scarce, due to lack of interest in performing and reporting these measurements. For example, most solids with poor ionic conductivity do not have conductivity data. The second challenge is that the parameterization of a ML model is highly susceptible to variances and errors in property data^38^. In the case of SSLCs, the conductivity obtained through experimental measurements can vary by a few orders of magnitude due to factors including synthesis method, sample preparation, and measurement technique^39^. For SSLCs, it is challenging to train a ML model of Li^+^ conductivity from only a few compounds with known values of *σ*_RT_ with large variances and to make reliable predictions for thousands of compounds. This scarcity of high-quality property data greatly limits the applicability of supervised ML models to capture and predict complex structure-property relationships over a broader space of materials beyond known examples.

Unlike supervised learning models, which require well-labeled training data, unsupervised learning can be readily applied to vast datasets regardless of whether any properties or labels exist. As a technique to draw inferences from features of data without explicitly labeled properties, unsupervised learning has been applied in materials science for feature extraction, pattern recognition, clustering, and phase mapping^40–44^. However, the application of unsupervised learning to directly discover new materials with enhanced properties has rarely been explored^27^. As shown in this study, unsupervised learning, through training on a broad range of materials, can draw boundaries between good and poor examples, identifying candidates similar to good examples, which are then further verified by more accurate first-principles calculations. This new approach using unsupervised learning for materials discovery has multiple advantages. Switching the target of ML from predicting the property (e.g., *σ*_RT_) in supervised learning to grouping materials in unsupervised learning alleviates the issues of poor data quality and accuracy. Rather than predicting the targeted materials property accurately for each candidate, the output from unsupervised learning is a significantly narrowed list of materials candidates for subsequent exploration by more accurate first-principles calculations, thus significantly reducing the cost for an expensive high-throughput first-principles screening by utilizing a limited quantity of low-quality data. In addition, unsupervised learning uses unlabeled data and readily expands the applicability of the ML model to the entire materials space.

In this study, we propose an unsupervised learning scheme for guiding materials discovery, and demonstrate it for materials discovery of SSLCs. We apply unsupervised learning to screen all known Li-containing compounds from the Inorganic Crystal Structure Database. Our trained unsupervised learning models cluster Li-containing compounds into groups of SSLCs with high conductivity and other groups of materials with poor ionic conduction. Using ab initio molecular dynamics (AIMD) simulation to quantify *σ*_RT_ for predicted compounds^45^, 16 new candidates having *σ*_RT_ exceeding 10^−4^ S cm^−1^ are identified, and three of them have *σ*_RT_ exceeding 10^−2^ S cm^−1^, on par with known SSLCs with highest *σ*_RT_. As proposed and demonstrated, our new approach of ML-guided materials discovery circumvents the data scarcity challenges, identifies new materials using a small number of known examples, and provides unique insight on structure-property relations.

## Results

### Scheme of the unsupervised discovery of SSLCs

We illustrate our scheme of the unsupervised discovery of SSLC materials in Fig. 1. In order to train the unsupervised model, a quantitative representation of the complex materials structure (Fig. 1a) is required as input. Instead of using a combination of hand-picked features, we used digital diffraction patterns of the crystal structure. Specifically, a representation for each crystal structure was built based on Bragg’s law to map the three-dimensional periodic crystal lattice into a set of X-ray diffraction intensities at a fixed set of 2*θ* values (Method and Fig. 1b)^35,46,47^. Here, we only considered the anion lattice of the crystal structure, relying on the knowledge that anion configuration and Li^+^-anion interactions significantly affect Li sites, diffusion channels, and the energy landscape of Li migration^1,13,15^. The anionic lattice was set to S anion and was scaled to the same atomistic volume, so that the representation was invariant to lattice parameter or the chemical constituent (Method). The resulting representation, called modified X-ray diffraction (mXRD), is unambiguously defined for every anion lattice (Fig. 1b), fully capturing the anionic crystal structure information. Here, we performed our unsupervised discovery on 2986 compounds that contain lithium but not transition metals. Since some compounds have the same structure, one representative structure was used. A dataset of 528 representative anionic structures and their mXRDs were performed for the unsupervised learning (Method).

### Unsupervised clustering of Li-containing compounds

**Fig. 2:**
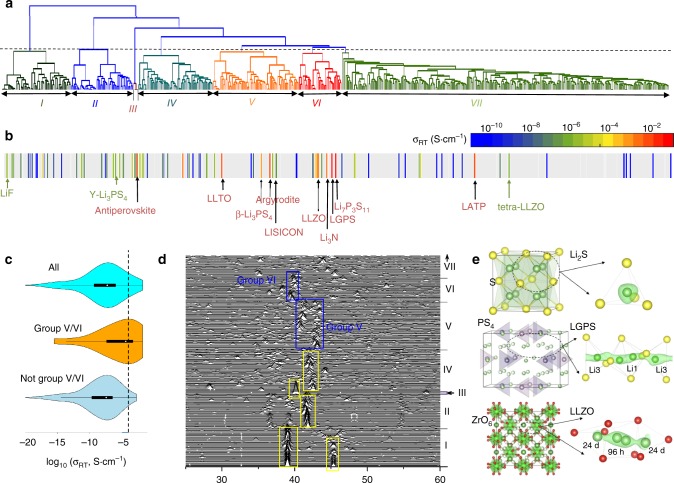
Unsupervised clustering of all Li-containing compounds. **a** Bottom-up tree diagram (dendrogram) generated using the agglomerative hierarchical clustering method. The dashed line shows the position where all compounds are partitioned into seven groups, marked as I–VII from left to right and distinguished by different colors. **b** Mapping the dendrogram to the conductivity reveals the grouping of known solid-state Li-ion conductors in group V and VI. The color bar shows the scale of *σ*_RT_. The gray color indicates the conductivity has not been measured for the corresponding compound. **c** Violin plots of *σ*_RT_ data grouped in the grouping. The outer shells of the violins bound all data, narrow horizontal lines bound 95% of the data, thick horizontal lines bound 50% of the data, and white dots represent medians. The dashed line shows the position of *σ*_RT_ = 10^–4^ S cm^−1^. **d** mXRD of all materials in group I–VI and a part of group VII. The colored boxes mark the positions of main characteristic peaks for each group. **e** Crystal structures (left) and (right) Li sites (green sphere) determined by local anion (yellow/red sphere) configuration, corresponding to isosurfaces (green) of Li probability density from AIMD simulations. Li_2_S (top) with highly symmetric anion lattice and ordered Li sublattice versus LGPS (middle) and LLZO (bottom) SSLCs with distorted anion lattices and disordered Li sublattices

We performed clustering, a common unsupervised learning technique, to group materials with similar mXRD representations. We first generated a model (named ***C***_***1***_) based on the agglomerative hierarchical clustering method to train a bottom-up grouping of the mXRD dataset (Method and Fig. 2a). The grouping showed a good quality of clustering as the mXRDs shared similar characteristics within the same groups (Fig. 2d) and different groups were well differentiated (Supplementary Note 1, Supplementary Fig. 1 and 2). More importantly, a visible clustering of SSLC materials is found using this model (Fig. 2b). Most known SSLCs with *σ*_RT_ close to 10^−3^–10^−2^ S cm^−1^, despite being structurally distinctive, were clustered into two groups in the center of the dendrogram out of a total seven groups, including LGPS, Li_7_P_3_S_11_, LLZO, and Li_3_N, in group VI, and argyrodite, β-Li_3_PS_4_, LLTO in group V. LATP, as an exception in group VII, lay close to the boundaries of group V and VII and its mXRD pattern still exhibited some similarity with group V. In addition, statistical analysis of *σ*_RT_ within the group quantitatively confirmed the correlation on *σ*_RT_ (Supplementary Note 1, Supplementary Figs 3 and 4). The violin plot of *σ*_RT_ of group V and VI showed significantly higher *σ*_RT_ (Fig. 2c), and the majority of compounds outside of group V and VI had *σ*_RT_ significantly below 10^−4^ S cm^−1^. A statistically significant difference of *σ*_RT_ of the two groups V and VI versus the rest groups was proved by the *t*-test (Supplementary Note 2).

As confirmed by quantitative correlation between the groups and *σ*_RT_, our unsupervised learning model captured the physical dependence of fast solid-state Li^+^-diffusion on anion lattice. To critically assess the robustness of clustering in capturing the observed physical correlation, we performed three different clustering techniques. In addition to the aforementioned model, we trained a second model (named ***C***_***2***_) to create a top-down grouping by recursively applying divisive spectral clustering (Method). These two models were purely based on the mXRD dataset of anion lattices without seeing any labeled *σ*_RT_ data. In our third grouping, the model (named ***C***_***3***_) used the limited available *σ*_RT_ information to optimize the clustering of known SSLC examples (Supplementary Note 4). Despite the differences in the clustering methodologies of three models, the observed aggregation of fast-conducting examples was mostly consistent. Known SSLCs largely overlapped among the groups generated by these three models (Supplementary Notes 3–5, Supplementary Table 1 and Supplementary Figs 5–9). In particular, LGPS, Li_7_P_3_S_11_, LLZO, and Li_3_N were always clustered to the same group by all three models. Our results from three distinct models confirmed the reliability of clustering fast-conducting versus poor-conducting materials based on unsupervised learning using mXRD representations of the structures.

### Physical insights from unsupervised learning

The clustering of SSLCs by mXRD provides new insight into the understanding of crystal structures exhibiting fast-ion conduction. While Li-ion diffusion in solids has been shown to correlate with various parameters, such as lattice volume^1^, anion chemistry^48^, bond ionicity^25,48^, phonon mode^16,17^, and Li coordination number^25^, no single unified theory explains the similarity among highly distinctive crystal structures of all SSLCs. Our unsupervised clustering quantitatively confirmed the similarity among the mXRD patterns of anion lattice of SSLCs. The mXRD encodes the symmetry and ordering of the anionic lattice and showed strong correlation with ionic conductivity (Fig. 2). Given the information of lattice volume and anion chemistry critical for ion diffusion were removed from the mXRD descriptor, the resulted clustering of Li-conducting phases suggests that the long-range periodicity of the anion lattice as encoded in mXRD plays a fundamental role in Li-ion diffusion. By analyzing the structural origin of the clustered groups, (Supplementary Note 6), we found the materials in Group I, II, and III correspond to highly symmetrical fcc (face centered cubic), hcp (hexagonal close packed), and bcc anion lattices, respectively. For these anion lattices, Li ions are symmetrically confined in highly symmetric tetrahedral or octahedral sites of anions (as an example, Fig. 2e for Li_2_S), and migrate among these well-defined sites^13^. Groups IV, V, and VI show a moderate level of variance, which can be understood as mild distortion of the anion lattices. The distortion of anion lattices disturbs Li^+^ bonding environments and causes Li^+^ to deviate from highly symmetric locations to geometrically frustrated configurations. For example, in LGPS and LLZO, the distorted anion polyhedra generate multiple positions to host Li ions, observed as the spread Li-ion probability density observed in AIMD simulations (Fig. 2e), which were represented as partially occupied Li sites (e.g., Li1 and 96 h sites in LGPS and LLZO, respectively) from diffraction experiments^4,5^. Having multiple positions for Li^+^ to occupy may lead to a degeneracy of Li sublattice energy and an entropically-enabled disordered-Li sublattice migrating among metastable configurations^18,19^. Therefore, as observed in their mXRD representations, the SSLCs clustered in group V and VI exhibit the characteristics of moderately distorted anion lattices, which is closely related to disordered Li sublattice for fast Li-ion conduction. The materials in Group VII, as reflected by the high standard deviation of mXRD peaks, correspond to the least symmetric and highly disordered anion lattices (Supplementary Figs 10–12). The highly disordered anion lattices in these materials may locally trap Li ions and impede Li-ion percolation across the crystal structure (Supplementary Fig. 13), resulting in the low conductivities observed for compounds in this group.

### SSLC confirmed by AIMD simulations

**Fig. 3:**
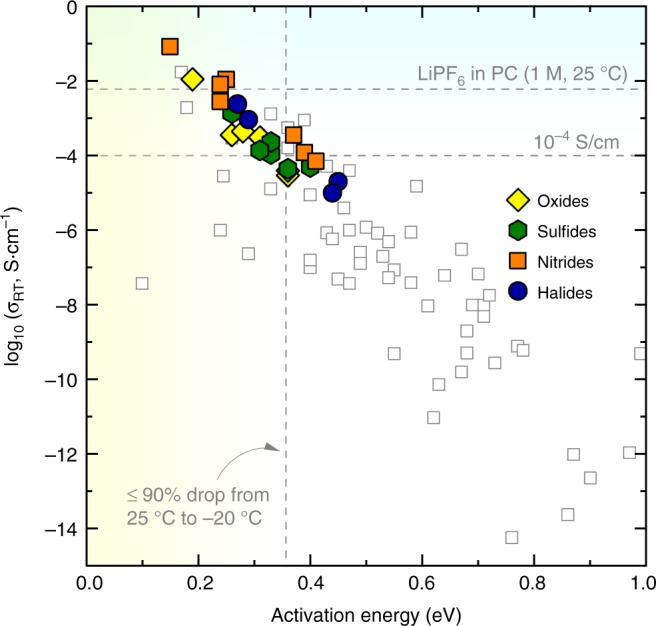
Ion conducting properties of newly predicted versus known solid-state Li-ion conductors. The open symbol shows the experimental conductivity reported in the literature (Supplementary Table 2 and references therein). The horizonal dashed lines show the room temperature conductivity of 10^–4^ and 0.006 S cm^−1^. The latter value is the conductivity of 1 M LiPF_6_ in propylene carbonate (PC) solution. The vertical dashed line shows the activation energy at 0.356 eV, which corresponds to one order of magnitude change of conductivity when the temperature drops from 25 to −20 °C

Given the successful clustering of known SSLC materials by unsupervised learning models, the other structures clustered into the same groups are expected to exhibit fast Li-ion conduction. To further assess the conductivity of these compounds discovered from the unsupervised grouping, we conducted AIMD simulations, which have been demonstrated as a highly accurate and predictive computation approach for calculating Li ion conductivity^11,14,15,45^. From the screening of initial 2989 compounds from the ICSD, we narrowed the evaluation of the ion-conduction property down to 82 unique compounds, which were from the intersection of these fast-conducting groups in the aforementioned three models. Thus, our unsupervised learning scheme successfully reduced a high throughput screening of thousands of compounds to a focused exploration of <100 candidates with much higher success probability. Among these, we rediscovered LiZnPS_4_, which was previously discovered by the bcc-anion-packing rule and was confirmed with an experimental *σ*_RT_ of 5.7 × 10^−4^ S cm^−1^^11–13^. According to AIMD simulations (Fig. 3), 16 more candidates are predicted to have *σ*_RT_ higher than 10^–4^ S cm^−1^. In particular, three new materials systems, Li_8_N_2_Se, Li_6_KBiO_6_ and Li_5_P_2_N_5_, have *σ*_RT_ exceeding 10^−2^ S cm^−1^, a conductivity higher than that of the best known SSLCs. A list of these materials and the calculated Li^+^ conduction properties are summarized in Supplementary Tables 3–4 and Supplementary Fig. 14. Figure 3 plots the predicted *σ*_RT_ and activation energy of newly discovered SSLCs (filled symbols), in comparison with *σ*_RT_ reported in the past few decades (open symbols, Supplementary Table 2). The newly discovered SSLCs are in the upper left corner of Fig. 3, which corresponds to high *σ*_RT_ of >10^−5^ S cm^−1^ and low *E*_a_ of 0.17–0.45 eV. More importantly, these SSLCs comprise new structures, chemistries, and compositions significantly different from known SSLCs, demonstrating the capability of our crystal-structure-based unsupervised learning model to discover materials beyond existing chemistries.

## Discussion

A fraction of compounds uncovered by our grouping did not show fast Li-ion diffusion in AIMD simulations (Supplementary Tables 5–7). Among these compounds, a majority exhibit too small of a percolation radius for Li-ion migration, a blocking of diffusion network by other cations, or a poorly connected diffusion network. The inclusion of these compounds was attributed to the fact that our unsupervised models were trained solely on the anionic geometry without considering factors such as the effects of other cations. In addition, some compounds with low ionic conductivity may be further optimized via doping or tuning Li concentration. Future extension of our scheme should attempt to include features in addition to the anion lattice for more accurate prediction.

For these Li-ion conductors to be utilized as solid electrolytes for solid-state Li-ion batteries, other materials properties, such as electrochemical window, interface compatibility, and mechanical properties^1,2,10,15,16^, are also required. We employed the first-principles computation techniques established in the previous studies^10,15^ to evaluate the thermodynamic intrinsic electrochemical window of these newly identified ion conductors (Supplementary Fig. 15). Consistent with the general trend identified in the previous studies^15^, most of the materials have limited electrochemical windows. Many identified nitrides are stable with Li metal in agreement with the previous computation study^49^, while other compounds are not stable against Li metal or at low potential due to the reduction of cations. The identified fluorides have a very high oxidation limit of >6 V, which may be ideal for stable protection of high-voltage cathodes. The oxides have decent electrochemical windows but most have relatively low ionic conductivities of ~10^−4^ S cm^−1^ (except for Li_6_KBiO_6_). The identified sulfides have narrow windows but these two sulfides may have significantly better air/moisture stability than currently used thio-phosphates. In summary, while our discovery does not identify an ionic conductor that out-competes current solid electrolytes, the potential choices of fast ion conductors with improvements in certain aspects (such as stability against Li metal, high voltage, or air) are predicted from the computation discovery. The properties and applicabilities of these materials in solid-state batteries may require further computational or experimental studies and optimizations.

In summary, the unsupervised learning models succeeded in distinguishing fast Li-conducting and poor Li-conducting materials, leading to the prediction of sixteen new compounds as solid-state Li-ion conductors with room-temperature conductivities higher than 10^−4^ S cm^−1^ with a few new compounds exceeding 10^−2^ S cm^−1^. These newly discovered candidates have highly different structures and chemical compositions from current known fast Li-ion conductors, demonstrating the effectiveness of our unsupervised learning approach for discovering new materials over a wide materials space. This novel unsupervised learning approach also reveals the unique structure–property relationship between anion lattice and Li^+^ conduction over a large materials space. Whereas the supervised learning has been widely adopted in the majority of machine-learning studies for materials, our unsupervised learning scheme, which narrows a high-throughput screening to a focused prioritized list by utilizing a limited amount of low-quality data, presents a different approach of using ML for materials discovery, and is generally applicable for other physical properties.

## Methods

### Data preprocessing

The raw data of crystalline structures were exported from the Inorganic Crystalline Structure Database (ICSD) in the format of *cif* files^50^. The range of analysis in the current study includes all compounds containing Li but not transition metal species, except Sc, Y, La, Ti, and Zr. The exclusion of transition metal species is based on the consideration that compounds containing transition metal ions are usually redox active and hence may not be suitable for application as solid-state electrolytes. These filters yielded a total of 2986 ICSD entries (ver. November 2016). The representative structures for each entry was identified either as the “chemical_name_structure_type” flag in the *cif* files or as the chemical formula if this flag was not set explicitly. The entries that were structurally similar in the hierarchical clustering were further filtered to remove duplicates in the training set. The final training set included 528 unique representative structures for the unsupervised learning analysis.

### Representation

The anionic sublattice of Li-containing compounds is uniquely represented in the X-ray diffraction pattern based on Bragg’s law. For the diffraction from (*hkl*) plane, the angle is determined by

1 $$2\sin \left( \theta \right) = \lambda \cdot d_{hkl}^{ - 1}$$

where the interplane distance *d*_*hkl*_ is a function of the size and shape of the unit cell

2 $$d_{hkl}^2 = h^2a^{ \ast 2} + k^2b^{ \ast 2} + l^2c^{ \ast 2} + 2hka^ \ast b^ \ast \cos \left( \gamma \right) + 2hla^ \ast c^ \ast \cos \left( \beta \right) + 2klb^ \ast c^ \ast \cos \left( \alpha \right)$$

The intensity is determined by the amplitude of light scattered from the lattice plane

3 $$F_{hkl} =\sum_{j = 1}^m {N_jf_j{\mathrm{exp}}\left[ {2\pi i(hx_i + ky_i + lz_i)} \right.}$$

where the sum runs over all atoms of the unit cell on (*hkl*) plane, *N*_*j*_ is the fraction of every equivalent position that is occupied by atom *j* at coordinates (*x*_*j*_, *y*_*j*_, *z*_*j*_). The scattering factor *f*_*j*_ is a product describing the interaction of the X-ray with the electrons around an atom. Using Eqs. 1, 2, and 3, the X-ray diffraction of a periodic lattice is determined by the size and shape of unit cell, as well as the position and identity of atoms on a given plane. The following procedure was employed to obtain the XRD representation of the geometry of anion sublattice of the crystalline structure. First, we removed all cations from the crystalline structure, keeping only the anionic sublattice in the unit cell. Second, we substituted the remaining anions for a unitary species (e.g., S^2−^), removing the influence of the scattering factor *f* on the diffraction intensity. Third, the unit cell was isotropically expanded or compressed to a pre-determined volume per anion of 40 Å^3^, removing the effect of unit cell size on the position of the diffraction peaks. After these initial steps, the X-ray diffraction of modified lattice encodes only information for the geometry and topology of anion sublattice. The calculation of diffraction pattern is then performed at a fixed set of 2*θ* values from 0 to 89.98^°^ at a step size of 0.1^°^ using the *pymatgen* package^51^, generating a 900-dimensional vector for each diffraction pattern^51^. We confirmed the results of hierarchical clustering was consistent when the step size was increased to 0.02^°^ (Supplementary Note 7). A Gaussian smearing was then performed to normalize the integrated intensity of diffraction to a unitary value.

### Unsupervised learning

We used the *Dendrogram* function from the *SciPy* package to perform agglomerative hierarchical clustering (AHC)^52^. In AHC, each sample starts in its own cluster, and the clusters merge progressively according to the similarity metric as one moves up the hierarchy. The output from AHC is a bottom-up hierarchical tree diagram (dendrogram). The Euclidean distance (L2) between two diffraction profiles was used as the similarity metric and Ward linkage was used to measure the cluster dissimilarity^53^. The same clustering results were also obtained using the *hclust* package in *R*.

In addition to the hierarchical clustering, we used the *kernlab* package in *R* to perform spectral clustering. Spectral clustering uses the eigenvalues of the similarity matrix of the data to divide the samples in to *K* groups, where *K* is a manually selected integer^54^. To create hierarchical grouping results, we recursively applied the bisectional divide (*K* = 2) on the larger portion from the previous grouping, and obtained a divisive top-down hierarchical diagram after the clustering.

### First-principles calculation

All Density Functional Theory (DFT) calculations were performed using the Vienna Ab initio Simulation package (VASP) within the projector augmented-wave approach and Perdew–Burke–Ernzerhof (PBE) generalized-gradient approximation (GGA) functionals^55–57^. The parameters in static DFT calculations were consistent with the *Materials Project*^58^. Ab initio molecular dynamics (AIMD) simulations were performed in supercell models using non-spin-polarized DFT calculations with a *Γ*-centered *k*-point. The time step was set to 2 fs. The initial structures were statically relaxed and were set to an initial temperature of 100 K. The structures were then heated to targeted temperatures at a constant rate by velocity scaling during 2 ps. During the estimation of Li ion diffusion, NVT ensemble using Nosé–Hoover thermostat was adopted. The total time of AIMD simulations were in the range of 100 ps to 1000 ps until the diffusivity was converged. The ionic diffusivity and conductivity were calculated following established method in previous study^45^.

## Supplementary information

Supplementary Information

## Data Availability

The diffraction data and AIMD simulation results are available through GitHub repository https://github.com/tri-na?tab=repositories. Other data generated during and/or analyzed during the current study are available from the corresponding authors on reasonable request.

## Code availability

The code used for the creation of unsupervised learning models used in the manuscript are available in the GitHub repository at https://github.com/tri-na?tab=repositories.
