# Supplementary material for: Unsupervised discovery of solid-state lithium ion conductors
Source: Nat Commun. 2019 Nov 20;10:5260. doi: 10.1038/s41467-019-13214-1 (PMC6868160; doi:10.1038/s41467-019-13214-1)
Supplement: Supplementary file 1 — Supplementary Information [file 41467_2019_13214_MOESM1_ESM.docx]

**Supplementary Information for**

**Unsupervised Discovery of Solid-state Lithium Ion Conductors**

Ying Zhang,^1^ Xingfeng He,^2^ Zhiqian Chen,^3^ Qiang Bai, ^2^ Adelaide M. Nolan,^2^ Charles A. Roberts, ^1^ Debasish Banerjee, ^1^ Tomoya Matsunaga, ^1^ Yifei Mo^2^ and Chen Ling^1^

1. Toyota Research Institute of North America, Ann Arbor, Michigan, 48105, United States
2. Department of Materials Science and Engineering, University of Maryland, College Park, Maryland 20742, United States
3. Department of Computer Science, Virginia Tech, 7054 Haycock Road, Falls Church, Virginia, 22043, United States
4. Maryland Energy Innovation Institute, University of Maryland, College Park, Maryland 20742, United States.

Correspondence and requests for materials should be addressed to Y. M. [yfmo@umd.edu](mailto:yfmo@umd.edu) and C. L. [chen.ling@toyota.com](mailto:chen.ling@toyota.com)

**Supplementary Note**

**Supplementary Note 1. Evaluate the quality of clustering.** We used the within-cluster variation to quantitatively evaluate the clustering quality in the mXRD space

$W=2\sum_{k=1}^{n_{G}} \sum_{i\in g_{k}} \left| {(mXRD}_{i}-\bar{mXRD}_{k}) \right|^{2}$ (1)

where $\bar{mXRD}_{k}=\frac{1}{|g_{k}|}\sum_{i\in g_{k}} {mXRD}_{i}$ is the average mXRD for group *k* with *|g_k_|* samples, *n*_G_ is the total number of groups and *g_k_* denotes group *k* in the clustering. Attributed to the apparent internal structure in the mXRD dataset (Supplementary Figure 1), the calculated *W* shown in Supplementary Figure 2 clearly distinguished the unsupervised clustering (***C_1_*** and ***C_2_***) with random clustering, suggesting a good quality of clustering in the mXRD space.

To evaluate the correlation between the clustering and the conduction property, we modify Eq. 4 to quantify the within-cluster variation on the *σ*_RT_ dataset

$W_{p}=2\sum_{k=1}^{n_{G}} \sum_{i\in g_{k}} {[{log(\sigma}_{RT})_{i}-{\bar{\log\left( \sigma_{RT} \right)}}_{k}]}^{2}$ (2)

where ${\bar{\log\left( \sigma_{RT} \right)}}_{k}$=$\frac{1}{{|g_{k}|}^{'}}\sum_{i\in g_{k}} {\sigma_{RT}}_{i}$ is the average log(σ_RT_) for group *k* with $|g_{k}|^{'}$ labeled samples. By this definition, a strong ***C***-σ_RT_ correlation is characterized by a small value of *W_p_* and vice versa.

While the AHC did not give an explicit number of group (*n*_G_) in the clustering, by defining the score function *W*_p_ we were able to determine the best number of groups in ***C_1_*** that yielded a good correlation with the conduction property. As shown in Supplementary Figure 3, the calculated *W*_p_ of ***C_1_*** changes from being comparable to that of random clustering to a sudden drop at a critical value of *n*_G_=7. Further analysis showed that this drop of *W*_p_ is not affected after adding a simulated noise to the conductivity data (Supplementary Figure 4). This critical number of groups *n*_G_=7 was then adopted in the analysis of ***C_1_*** results, which corresponded to cut the dendrogram at the level shown in Figure 2a in the manuscript.

**Supplementary Note 2. *t*-test of the clustering of conductivity.** We performed the student’s *t*-test to examine the statistical difference of conductivity in different groups in ***C_1_***. Given the observation that most known conductors were grouped in either V or VI, we set the null hypothesis as the conductivity in these two groups had no difference than other groups. It can be formulated

$H_{0}: \sigma_{RT,i}-\sigma_{RT,j}=0$ (3)

$H_{1}:\left| \sigma_{RT,i}-\sigma_{RT,j} \right|>0$ (4)

where *i* and *j* denote samples in and out of group V/VI, respectively.

The p-score to compare the conductivities in group V/VI samples with those not in group V/VI was 0.0046, not assuming an equivalent variance. In addition, the p-score to compare the conductivities in group V/VI samples with those in group I/II was 0.0235, not assuming an equivalent variance. Both tests rejected the null hypothesis at 95% confidence level, proving the results of ***C***_1_ has a statistical correlation with the *σ*_RT_ data.

**Supplementary Note 3. Compare *C_1_* and *C_2_* results.**

In the current study the ***C_1_*** was created by applying the AHC on all samples, while the ***C_2_*** was created by recursively applying the spectral clustering on all samples. Whereas these two methods differ in the clustering algorithm and similarity measurement, the results of ***C_1_*** and ***C_2_*** were in fact highly similar. As shown in Supplementary Figure 5, ***C_2_*** first divided out a small group, which contained the same samples as that of group III in ***C_1_***. In the second layer, a group was separated along the first principle component direction, which corresponded to group I in ***C_1_***. In the third and fourth layer, a group corresponding to group II and IV in ***C_1_*** is separated, respectively. The known SSLCs appear in the 5^th^ layer including Argyrodite, LLTO and LISICON, and the 6^th^ layer including LLZO, Li_3_N, LGPS and Li_7_P_3_S_11_. The β-Li_3_PS_4_ is grouped into the 7^th^ layer and NASICON goes into the remaining group.

**Supplementary Note 4. Creation and results of *C_3_*.**

The selection of the subset of mXRD representation was treated as decreasing the score function *W*_p_ through optimizing a 900-dimensional vector ***v*** with *v*_j_=1 or 0 that determines the inclusion or exclusion of the diffraction intensity at a special *θ* value. We used the coordinate descent method to perform the optimization, which was implemented in a customer written code. To improve the stability of the optimization and avoid the oversimplified feature representation, we define two *W*_p_ functions to measure the clustering of conductivity for the full samples, and for the samples of group IV to VII in ***C_1_***. Starting from the original diffraction data (***v***=**1**), the exclusion of diffraction intensity at *θ*_j_ (*v*_j_=0) was determined only if it does not increase any of the objective functions. The evolution of the dimensionality of the selected subset is shown in Supplementary Figure 6. After 8 iterations of optimization, the dimensionality was reduced to 443 and the results did not change in the next three iterations. Using the optimized ***v*** from the 8^th^ iteration, the subset of mXRD is selected as

$I_{j}^{'}=I_{j}\cdot v_{j}$ (5)

where $I_{j}$ and $I_{j}^{'}$ is the diffraction intensity in the original mXRD and in the subset, respectively.

We then performed the hierarchical clustering based on the selected subset to group all samples and the samples of group IV to VII in ***C_1_***. The calculated *W*_p_ was shown in Supplementary Figure 7. For the clustering of group IV to VII samples (***C_3_***), the best conductivity-correlated clustering had five groups. Supplementary Figure 8 shows the results of ***C_3_*** in the principle component space of the mXRD subset. Detailed analysis shows that the results of ***C_3_*** can be described as a group identical to group IV in ***C_1_***, the re-organization of group V, VI and a small portion of VII samples in three groups, and a group containing the rest of group VII samples. The known SSLCs appeared in the groups from re-organized V, VI and VII samples. Despite seeing the *σ*_RT_ information in the clustering, the result of ***C_3_*** is still highly similar to that of ***C_1_***, further proving the robustness of the observed ***C***-*σ*_RT_ correlation.

**Supplementary Note 5. Compare *C_1_*, *C_2_* and *C_3_* results.**

Supplementary Figure 9 shows the distribution of samples in the groups containing known SSLCs in ***C_1_*, *C_2_*** and ***C_3_*** models. We notice these three sets largely overlap with each other. To quantitatively assess the similarity between these groups, we calculate the overlap coefficient in the Szymkiewicz–Simpson way as the size of the [intersection](https://en.wikipedia.org/wiki/Intersection_(set_theory)) divided by the smaller of the size of the two sets^1^

$O_{X,Y}=\frac{size(X\cap Y)}{min[size\left( X \right),size\left( Y \right)]}$ (6)

The overlap coefficients are reported in Supplementary Table 1. We found for compounds with ordered fcc, hcp and bcc anion sublattice, the three models gave the overlap coefficient over 90%. For the prioritized group, the overlap coefficient is 78% for *C_1_* and *C_2_*, 93% for *C_1_* and *C_3_*, 85% for C_2_ and C_3_. The overlapped portion among three groups is 74.5%. These large overlap coefficients demonstrate the robustness of our clustering results.

**Supplementary Note 6. *C_1_* clustering of mXRD and** **correlation to σ_RT_**

Here we analyze the mXRD patterns of all materials (528×900 matrix) in ***C_1_*** to understand the strong correlation of ***C_1_*** groups on σ_RT_ discovered in our study. Through studying the average of mXRD spectra in each group, we show highly symmetric lattices in group I, II and III, lower symmetric lattice in group IV, V and VI, and the lowest symmetric lattices in group VII.

**Group I, II and III:** As shown in Supplementary Figure 10, the averaged mXRD for these three groups are characterized by sharp and strong peaks. The high intensity of averaged mXRD is thus a result of the high symmetry of these three prototype lattices as well as the facts that the grouped samples all have similar anion lattices, resulting in a low standard deviation of the averaged spectra. These spectra match well to the diffraction of fcc, hcp and bcc lattices for group I, II and III, respectively, suggesting materials in these three groups are composed of compounds with ordered or slightly distorted fcc-, hcp- and bcc-type anionic sublattices. For example, group I includes rocksalt-type LiF and spinel LiAl_2_O_4_, both of which are well-known compounds with closed packed fcc oxygen-lattice. Group II includes olivine LiMgPO_4_, a structure with closed packed hcp oxygen-lattice. In agreement with the report of Wang et al., we found that γ-Li_3_PS_4_ and Li_4_GeS_4_ are also classified as hcp-sublattice, although the latter seems to be positioned at the boundary between group II and group IV. Including antiperovskite Li_3_OCl, only five compounds are classified into group III with ordered bcc-packing. These results suggest that the unsupervised learning is capable to identify compounds with the three prototypes of highly symmetric lattices.

**Group IV, V and VI:** As shown in Supplementary Figure 9, weaker peaks with much wider standard deviation are displayed in the average spectrum of group IV to VI. This observation is a result of (i) less symmetric lattices and (ii) more than one prototypes of lattices within the same group. Here we reveal the clustering principle for these three groups. In Figure 2d, we plot all the mXRD spectra on the same order as in the ***C_1_*** clustering. Some patterns are visible, as marked in Figure 2d. Therefore, although multiple types of lattices are included in each of these three groups, the mXRD representations still exhibit patterns captured by our unsupervised clustering. These patterns of the clustered compounds result in the low within-cluster variation of mXRD data (Supplementary Figure 2). As shown in Figure 2d, the individual mXRD spectrum is also noisier compared to group I to III, suggesting lower symmetric lattices in these three groups.

**Group VII:** The average spectrum of group VII has no appreciable peak (Supplementary Figure 10). This noisy characteristic persists in individual mXRD spectrum as seen in Supplementary Figure 11. It is interpreted as this groups contains a large quantity of lattice having the largest distortion.

With the above results, we conclude that the general trend to group Li-containing compounds in ***C_1_*** is to sort the symmetry of anionic geometry from high to low when moving from left to right. To further quantitatively confirm this result, we calculate the standard deviation ($SD$) of mXRD spectra. For highly symmetric lattices such as fcc, hcp and bcc, the spectra are characterized by a few sharp peaks from the diffraction on degenerate planes and hence have large values of $SD$. On the other side, small $SD$ is expected for the noisy spectra of lattices with low symmetry. In the extreme case of diffraction for an amorphous solid, the spectrum contains no major peak and hence the $SD$ should be close to zero. As shown in Supplementary Figure 12, the violin plot of the calculated distribution of $SD$ exhibited a shift toward lower values from group I to group VII compounds, clearly demonstrating the ordering of symmetry in ***C_1_***. A large portion of Li-containing compounds have $SD$ below 0.03. The $SD$s of all known SSLCs lie between 0.032 and 0.062. For Li_1.3_Al_0.3_Ti_1.7_(PO_4_)_3_ (LATP), which is classified in group VII, the calculated $SD$ is 0.035. Therefore, the results of ***C_1_*** show that the anionic sublattices of all known SSLCs have intermediate symmetry neither as high as fcc or hcp nor as low as those in group VII.

**Supplementary Note 7. Influence of diffraction step size in the clustering results**

We selected a step size (Δ2θ) of 0.1° and simulated the diffraction intensity from 0 to 89.9°. It yielded a 900-dimensional vector to represent the anionic sublattice. We selected a various of different step sizes and found the hierarchical clustering (*C*_1_) was invariant when Δ2θ changed from 0.1° to 0.02°. Following the same procedure, we performed the spectral clustering (*C*_2_) and the hierarchical clustering using an optimized subset of mXRD (*C*_3_) using a 4500-dimensional representation (Δ2θ =0.02°) and obtained a second list of potential candidates for Li-ion conductors. Comparing the results from Δ2θ =0.1° and 0.02° gave an overlap coefficient of 87% for the good candidate list. Moreover, the 16 identified candidates with conductivity higher than 0.1 mS/cm (Supplementary Table 3) all appeared in both results. For those with conductivities between 0.01 to 0.1 mS/cm (Supplementary Table 4), Li_6_Si_2_O_7_ (0.04 mS/cm) only appeared in the 0.1° result, and a new candidate (Li_3_BN_2_, 0.07 mS/cm) was found in the 0.02° result. The rest six compounds were included in both results. Thus, we concluded that the unsupervised learning yielded a list of candidates consistent to the choice of step size from 0.1° to 0.02° in representing the diffraction pattern.

**Supplementary Table 1. Overlap coefficient between three classification models.**

|  | fcc | hcp | bcc | G4 | G5/6 (prioritized) | G7 |
| --- | --- | --- | --- | --- | --- | --- |
| C1&C2 | 100% | 93.9% | 100% | 71.2% | 78.4% | 92.6% |
| C1&C3 | 100% | 100% | 100% | 89.8% | 93.1% | 94.8% |
| C2&C3 | 100% | 93.9% | 100% | 67.2% | 84.9% | 93.6% |
| C1&C2&C3 | 100% | 93.9% | 100% | 64.4% | 74.5% | 91.5% |

**Supplementary Table 2.** Dataset of Experimental ionic conductivity (*σ*_RT,_ S/cm) at room temperature and activation energy (*E*_a_, eV) from available literature reports of conductivity measurements. For each structure prototype, the highest reported value is used as the representative example. For those measurements not performed at room temperature (300 K), we extrapolate the *σ*_RT_ using the Arrhenius relation. Note that this dataset is only used in the training of ***C_3_*** model and in quantifying the correlation of ***C*_1_** to *σ*_RT_, whereas the training of ***C_1_*** and ***C_2_*** does not utilize this dataset.

| Compound | *σ*_RT_ | *E*_a_ | reference |
| --- | --- | --- | --- |
| α-Li_3_BN_2_ | 1.60E-10 | 0.67 | 2 |
| γ-Li_3_PS_4_ | 2.60E-07 | 0.49 | 3 |
| β-Li_3_BN_2_ | 8.70E-08 | 0.55 | 2 |
| β-Li_3_PS_4_ | 1.60E-04 | 0.36 | 3 |
| Li_6_PS_5_Cl | 1.30E-03 | 0.33 | 4 |
| Li_0.87_Hf_2.032_P_3_O_12_ | 1.29E-05 | 0.33 | 5 |
| La_0.5_Li_0.5_TiO_3_ | 9.00E-04 | 0.39 | 6 |
| Li_10_GeP_2_S_12_ | 1.20E-02 | 0.25 | 7 |
| Li_14_Zn(GeO_4_)_4_ | 1.00E-06 | 0.24 | 8 |
| Li_2.667_Mg_0.667_P_2_S_6_ | 4.00E-06 | 0.46 | 9 |
| Li_2_B_3_PO_8_ | 5.80E-15 | 0.76 | 10 |
| Li_2_B_4_O_7_ | 1.00E-10 |  | 11 |
| Li_2_MgBr_4_ | 7.80E-10 | 0.77 | 12 |
| Li_2_NaBP_2_O_8_ | 4.40E-18 | 1.21 | 10 |
| Li_2_SiN_2_ | 1.60E-07 |  | 13 |
| Li_2_SnS_3_ | 1.50E-05 | 0.59 | 14 |
| Li_2_SO_4_ | 1.40E-14 | 1.1 | 15 |
| Li_2_ZnGeO_4_ | 1.00E-07 | 0.4 | 16 |
| Li_2_ZnI_4_ | 4.00E-08 | 0.58 | 17 |
| Li_2_ZrO_3_ | 6.10E-10 | 0.78 | 18 |
| Li_3_AlN_2_ | 5.00E-08 | 0.45 | 19 |
| Li_3_BO_3_ | 7.40E-11 | 0.63 | 20 |
| Li_3_BP_2_O_8_ | 9.60E-12 | 0.62 | 10 |
| Li_3_N | 3.00E-04 | 0.26 | 21 |
| Li_3_Na_5_(TiS_4_)_2_ | 8.80E-06 | 0.4 | 22 |
| Li_3_OBr | 1.94E-03 | 0.18 | 23 |
| β-Li_3_PO_4_ | 3.00E-16 | 1.14 | 24 |
| Li_3_SbS_3_ | 1.00E-07 | 0.4 | 25 |
| Li_4_GeO_4_ | 2.80E-10 | 0.73 | 26 |
| Li_4_GeS_4_ | 2.00E-07 | 0.53 | 27 |
| Li_4_P_2_S_6_ | 2.38E-07 | 0.29 | 28 |
| Li_4_SiO_4_ | 5.00E-10 | 0.55 | 29 |
| Li_4_SiS_4_ | 5.00E-08 |  | 30 |
| Li_4_SrN_2_ | 2.30E-13 | 0.9 | 31 |
| Li_5_AlO_4_ | 5.00E-10 | 0.99 | 32 |
| Li_5_GaO_4_ | 5.00E-09 | 0.71 | 32 |
| Li_5_La_3_Bi_2_O_12_ | 4.00E-05 | 0.47 | 33 |
| Li_5_NCl_2_ | 1.20E-06 | 0.5 | 34 |
| Li_6.5_O_8_P_1.5_Si_0.5_ | 1.30E-07 | 0.49 | 35 |
| Li_6_Ge_2_O_7_ | 8.50E-07 | 0.43 | 36 |
| Li_6_La_2_SrBi_2_O_12_ | 5.20E-05 | 0.43 | 33 |
| Li_6_NBr_3_ | 1.00E-08 | 0.69 | 37 |
| Li_6_NI_3_ | 3.70E-06 |  | 38 |
| Li_6_ZnO_4_ | 9.40E-09 | 0.61 | 32 |
| Li_6_Zr_2_O_7_ | 5.20E-10 | 0.68 | 39 |
| Li_7_BiO_6_ | 8.80E-07 | 0.58 | 40 |
| Li_7_P_3_S_11_ | 1.70E-02 | 0.17 | 41 |
| Li_7_PN_4_ | 1.60E-07 | 0.4 | 42 |
| Li_7_SbO_6_ | 6.70E-08 | 0.7 | 40 |
| Li_9_NS_3_ | 8.30E-07 | 0.52 | 43 |
| LiAlCl_4_ | 1.00E-06 | 0.47 | 44 |
| Li_3_AlF_6_ | 5.00E-07 | 0.54 | 45 |
| LiAlSi_3_O_8_ | 1.30E-10 |  | 46 |
| LiAlSi_4_O_10_ | 1.01E-10 |  | 46 |
| LiAlSiO_4_ | 2.00E-09 | 0.68 | 47 |
| LiB_6_O_9_F | 5.40E-24 | 1.38 | 48 |
| LiBaP_2_O_7_ | 1.00E-10 |  | 49 |
| LiBiO_2_ | 3.80E-08 | 0.1 | 20 |
| LiBO_2_ | 1.00E-08 | 0.71 | 50 |
| LiCdCl_4_ | 5.80E-07 | 0.44 | 51 |
| LiGa_2_GeS_6_ | 3.80E-08 | 0.47 | 52 |
| LiGaO_2_ | 2.40E-14 | 0.86 | 53 |
| LiGaSiO_4_ | 3.00E-16 | 0.9 | 54 |
| LiIO_3_ | 1.90E-07 |  | 55 |
| LiMgSO_4_F | 5.40E-08 | 0.54 | 56 |
| LiNaSO_4_ | 8.80E-10 |  | 57 |
| LiPO_3_ | 1.00E-09 |  | 58 |
| LiPN_2_ | 1.6E-7 | 0.40 | 42 |
| LiScO_2_ | 1.00E-12 | 0.87 | 18 |
| LiSi_2_N_3_ | 6.17E-08 | 0.64 | 59 |
| LiYO_2_ | 1.80E-08 | 0.72 | 18 |
| LiZnSO_4_F | 2.80E-05 | 0.2455 | 60 |
| LLZO | 5.60E-04 | 0.36 | 61 |
| r-LiAlO_2_ | 1.10E-12 | 0.97 | 18 |
| tetragonal-LLZO | 3.10E-07 | 0.67 | 62 |

**Supplementary Table 3. Ionic conductivity of 16 new solid-state Li-ion conductors (***σ*_RT_**>0.1 mS/cm) predicted by AIMD simulation.** The table contains the information of ICSD number, the stoichiometric composition (Comp.), the doped composition in AIMD simulation (doped comp.), the energy of the doped compound above the thermodynamic convex hull (*E*_hull_, meV/atom), the activation energy of diffusivity (*E*_a_, eV), the preexponential factor of the diffusivity (*D*_0_, cm^2^/s), the predicted RT conductivity (*σ*_RT_, mS/cm) and the error range of the predicted conductivity ([*σ*_min_, *σ*_max_], mS/cm).

| ICSD | Comp. | doped comp. | *E*_hull_ | *E*_a_ | log(*D*_0_) | *σ* | [*σ*_min_, *σ*_max_] |
| --- | --- | --- | --- | --- | --- | --- | --- |
| 51754 | Li_3_Al(BO_3_)_2_ | Li_2.5_Al(B_0.75_C_0.25_O_3_)_2_ | 46 | 0.26±0.05 | -9.39±0.67 | 0.36 | [0.04, 3.2] |
| 71058 | Li_5_P_2_N_5_ | Li_5_P_2_N_5_ | 0 | 0.15±0.01 | -8.98±0.14 | 82.4 | [53.9, 126.0] |
| 18313 | Li_2_TiF_6_ | Li_3_TiOF_5_ | 64 | 0.29±0.02 | -7.76±0.28 | 0.92 | [0.38, 2.24] |
| 71035 | Li_6_KBiO_6_ | Li_6.5_KBi_0.5_Sn_0.5_O_6_ | 22 | 0.19±0.02 | -9.51±0.32 | 11 | [2.1, 15.7] |
| 409384 | Li_3_GaF_6_ | Li_3.75_Ga_0.75_F_6_ | 30 | 0.27±0.03 | -7.81±0.30 | 2.4 | [0.83, 6.7] |
| 262642 | Li_2_In_2_SiS_6_ | Li_2.75_In_1.75_SiS_6_ | 67 | 0.26±0.03 | -7.85±0.39 | 1.4 | [0.21, 9.3] |
| 247255 | Li_8_N_2_Se | Li_7.5_N_1.5_Se_1.5_ | 45 | 0.25±0.02 | -7.58±0.23 | 10.9 | [5.7, 20.8] |
| 247257 | Li_8_N_2_Te | Li_7.75_N_1.75_Te_1.25_ | 29 | 0.39±0.01 | -6.54±0.11 | 0.12 | [0.04, 0.36] |
| 67536 | LiSiBO_4_ | Li_1.375_SiB_0.875_O_4_ | 46 | 0.31±0.05 | -7.84±0.50 | 0.31 | [0.02, 4.4] |
| 34256 | LiBO_2_ | Li_0.75_Mg_0.125_BO_2_ | 58 | 0.28±0.04 | -9.02±0.31 | 0.45 | [0.09, 2.3] |
| 419061 | LiAsS_2_ | Li_1.1875_As_0.9375_S_2_ | 52 | 0.33±0.02 | -7.73±0.31 | 0.11 | [0.02, 0.52] |
| 92312 | Li_3_Na_3_N_2_ | Li_2.78_Mg_0.11_N | 15 | 0.24±0.03 | -8.41±0.50 | 8.1 | [2.1, 31.3] |
| 78819 | Li_10_BrN_3_ | Li_9.17_BrSe_0.83_N_2.17_ | 68 | 0.37±0.02 | -6.23±0.29 | 0.36 | [0.17, 0.80] |
| 84649 | Li_4_NCl | Li_3.56_Mg_0.22_NCl | 34 | 0.24±0.04 | -8.89±0.37 | 2.8 | [0.17, 44.6] |
| 429902 | Li_17_Sb_13_S_28_ | Li_5_Sb_3_S_7_ | 39 | 0.33±0.03 | -7.16±0.32 | 0.23 | [0.01, 8.5] |
| 40457 | LiSbS_2_ | Li_0.89_Mg_0.055_SbS_2_ | 47 | 0.31±0.01 | -8.15±0.09 | 0.14 | [0.07, 0.29] |

**Supplementary Table 4. Ionic conductivity of 7 Li-containing compounds with 0.01 mS/cm** *< σ*_RT_ **< 0.1 mS/cm predicted by AIMD simulation.** The table contains the information of ICSD number, the stoichiometric composition (Comp.), the doped composition in AIMD simulation (doped comp.), the energy of the doped compound above the thermodynamic convex hull (*E*_hull_, meV/atom), the activation energy of diffusivity (*E*_a_, eV), the preexponential factor of the diffusivity (*D*_0_, cm^2^/s), the predicted RT ionic conductivity (*σ*_RT_, mS/cm) and the error range of the predicted conductivity ([*σ*_min_, *σ*_max_], mS/cm).

| ICSD | Comp. | Doped comp. | *E*_hull_ | *E*_a_ | log(*D*_0_) | *σ* | [*σ*_min_, *σ*_max_] |
| --- | --- | --- | --- | --- | --- | --- | --- |
| 85171 | Li_3_AlF_6_ | Li_3.75_Al_0.75_F_6_ | 41 | 0.45±0.04 | -5.76±0.52 | 0.02 | [0.004, 0.08] |
| 9987 | Li_3_Ga(BO_3_)_2_ | Li_2.5_Ga(B_0.75_C_0.25_O_3_)_2_ | 62 | 0.36±0.02 | -8.34±0.16 | 0.03 | [0.01, 0.13] |
| 262643 | Li_2_In_2_GeS_6_ | Li_2.75_In_1.75_GeS_6_ | 73 | 0.36±0.01 | -7.17±0.07 | 0.045 | [0.02, 0.09] |
| 84763 | Li_5_Cl_2_N | Li_4.67_Cl_2_Se_0.33_N_0.67_ | 34 | 0.41±0.04 | -6.01±0.52 | 0.07 | [0.01, 0.32] |
| 608360 | LiAlS_2_ | Li_1.5_Al_0.5_Be_0.5_S_2_ | 63 | 0.40±0.04 | -6.20±0.63 | 0.05 | [0.009, 0.34] |
| 18004 | Li_6_ZrBeF_12_ | Li_7_ZrBe_0.5_F_12_ | 33 | 0.44±0.04 | -6.22±0.62 | 0.01 | [0.002, 0.08] |
| 25752 | Li_6_Si_2_O_7_ | Li_6_Si_2_O_7_ | 0 | 0.36±0.14 | -3.83±0.76 | 0.04 | [0.0001, 9] |

**Supplementary Table 5. Ionic conductivity of five Li-containing compounds with** *σ*_RT_ < **0.01 mS/cm predicted by AIMD simulation.** The table contains the information of ICSD number, the stoichiometric composition (Comp.), the doped composition in AIMD simulation (doped comp.), the energy of the doped compound above the thermodynamic convex hull (*E*_hull_, meV/atom), the activation energy of diffusivity (*E*_a_, eV), the preexponential factor of the diffusivity (*D*_0_, cm^2^/s), the predicted conductivity (*σ*_RT_, mS/cm) and the error range of the predicted conductivity ([*σ*_min_, *σ*_max_], mS/cm).

| ICSD | Comp. | Doped comp. | *E*_hull_ | *E*_a_ | log(*D*_0_) | *σ* | [*σ*_min_, *σ*_max_] |
| --- | --- | --- | --- | --- | --- | --- | --- |
| 87414 | LiSrN | Li_1.2_Sr_0.9_N | 29 | 0.70±0.10 | -5.79±1.00 | 8.1×10^-7^ |  |
| 402341 | LiBeN | Li_0.88_Be_0.94_Ge_0.06_N | 62 | 0.76±0.15 |  | 1.2×10^-7^ |  |
| 79352 | LiZnPO_4_ | Li_1.5_Zn_0.75_PO_4_ | 9 | 0.63±0.13 | -5.13±1.11 | 1.6×10^-5^ |  |
| 67535 | LiGeBO_4_ | Li_1.1875_GeB_0.9375_O_4_ | 24 | 0.74±0.15 | -6.59±1.3 | 5.1×10^-8^ |  |
| 23406 | Li_2_GeF_6_ | Li_3_Ge_0.75_F_6_ | 64 | 0.61±0.13 | -3.48±1.75 | 4.0×10^-4^ | [1.5×10^-6^, 0.09] |
| 67264 | Li_7_Br_3_O_2_ | Li_6_Mg_0.5_Br_3_O_2_ | 47 | 0.46±0.03 | -7.97±0.25 | 1.4×10^-3^ | [3.2×10^-5^,0.07] |

**Supplementary Table 6. Compounds that showed poor conductivity in AIMD simulation.** Five compounds showed with poor diffusion (low *D*) at 1150 K, two have high activation energy (high *E*_a_) barrier estimated from two AIMD simulations at 1150 and 900 K, and three have the melting of the crystal structure in the AIMD simulation conditions.

| ICSD | Comp. | reasoning |
| --- | --- | --- |
| 424835 | Li_3_AsS_3_ | low *D* |
| 246302 | Li_2_SeO_4_ | low *D* |
| 26297 | Li_6_TeO_6_ | melting |
| 180011 | Li_0.5_TiO_2_ | High E_a_ |
| 92395 | Li_4_SeO_5_ | low *D* |
| 59640 | Li_4_ZnP_2_O_8_ | low *D* |
| 171375 | LiBF_4_ | melting |
| 200357 | Li_0.6_Si_1.3_Zn_1.1_O_4_ | melting |
| 9430 | LiBe_2_Na_2_F_7_ | melting |
| 32713 | LiPN_2_ | Low D |
| 68463 | Li_4_Ge_5_O_12_ | melting |
| 153522 | LiTiOPO_4_ | low *D* |
| 172581 | LiTiOAsO_4_ | low *D* |

**Supplementary Table 7. Compounds that were excluded without performing AIMD simulation.**

| ICSD | Comp. | reasoning |
| --- | --- | --- |
| 27672 | LiK_2_AlF_6_ | small radius |
| 39669 | Li_1.39_K_2_Y_0.87_F_6_ | Same as above |
| 27318 | LiBa_4_Sb_3_O_12_ | small radius |
| 39604 | LiKBaZnF_6_ | small radius |
| 15642 | Li_2_BaSi | silicide |
| 99503 | Li_4_CaB_2_O_6_ | 2D network |
| 416101 | Li_7.23_B_7_Se_15_ | selenide |
| 93540 | Li_2_Ti_3_Bi_4_O_12_ | bad network |
| 14360 | Li_2_BeF_4_ | 2D network |
| 30982 | LiAlSiO_4_ | 2D network |
| 65126 | LiAlGeO_4_ | 2D network |
| 65127 | LiGaGeO_4_ | 2D network |
| 81857 | LiLaTiO_4_ | small radius |
| 89903 | LiSb_3_O_8_ | 1D network |
| 236294 | Li_2_MgBa(PO_4_)_2_ | Mg blocking |
| 29668 | LiAlSi_2_O_6_ | bad network |
| 280108 | LiGaSi_2_O_6_ | bad network |
| 193803 | Li_2_Ti_3_O_7_ | 1D network |
| 202439 | LiAlLa_4_O_8_ | small r |
| 100516 | LiBa_3_Ti_5_Sb_3_O_21_ | small r |
| 427078 | Li_2_Mg_2_Ca_2_Si_2_N_6_ | bad network |
| 182966 | Li_2_Ti_6_O_13_ | 1D network |
| 411410 | Li_2_B_2_Se_5_ | selenide |
| 189825 | LiTiS_2_ | 2D network |
| 28388 | LiGaO_2_ | 2D network |
| 422680 | LiCNO | CN anion |
| 44957 | LiYS_2_ | 2D network |
| 415120 | Li_2_TeS_3_ | bad network |
| 162953 | Li_2_MgSi_5_O_12_ | Mg blocking |
| 27007 | Li_3_Na_3_Sc_2_F_12_ | small radius |
| 27008 | Li_3_Na_3_In_2_F_12_ | small radius |
| 30253 | Li_3_Na_3_Al_2_F_12_ | small radius |
| 401208 | LiK_2_GaAs_2_ | pnictide |
| 402083 | LiNa_2_AlP_2_ | pnictide |
| 402111 | LiNa_2_GaAs_2_ | pnictide |
| 402147 | LiK_2_InAs_2_ | pnictide |
| 35676 | LiGeTe_2_ | telenide |
| 32028 | LiYSi | silicide |
| 32029 | LiYGe | Ge anion |
| 25105 | Li_3_Ba_2_Ti_9.25_O_22_ | Ti blocking |


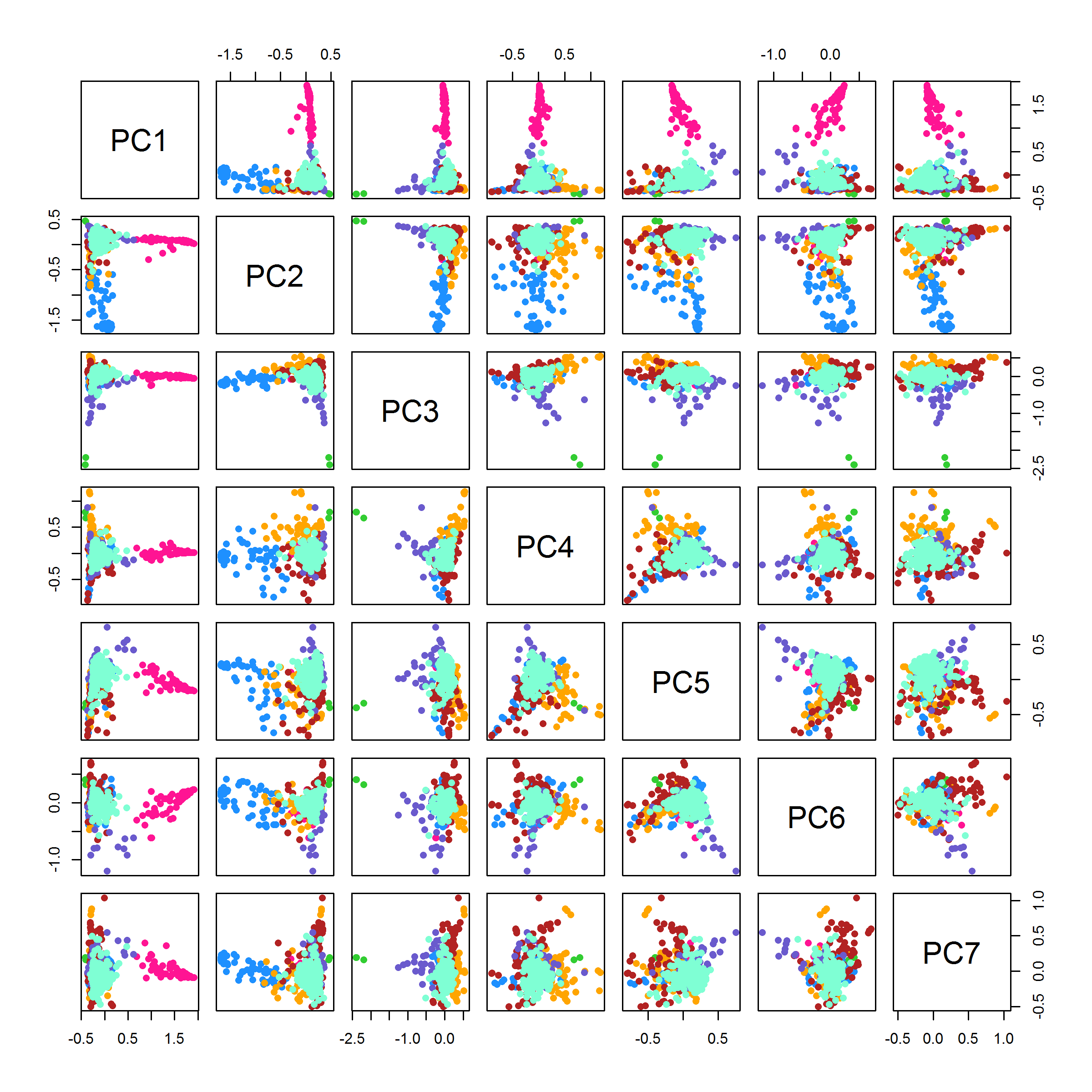


**Supplementary Figure 1. Visualization of mXRD samples in the principle component (PC) space.** We used the results from the ***C_1_*** model to color each sample (group I: pink; II: light blue; III: green; IV: orange; V: wine; VI: light purple; VII: cyan). The most apparent internal structure is the two arms extended along the first and second PC directions. Some patterns on higher ranked PC directions are also distinguishable such as the isolation of group III and V on the third and fifth PC direction, respectively.





**Supplementary Figure 2. The within-cluster variation (*W*) of the random clustering, the hierarchical clustering (*C_1_*) and the spectral clustering (*C_2_*).** The values were normalized by the averaged *W* of random clustering. The random clustering was performed 100 times by dividing samples randomly into a given number of groups. As expected, the random clustering lacked the power of clustering similar samples, characterized by the calculated *W* nearly independent of the number of groups (*n*_G_). In contrast, both ***C_1_*** and ***C_2_*** had W monotonically decreased with *n*_G_. For *n*_G_≥7, *W* decreased by more than 30%, proving a good quality of clustering in the unsupervised clustering.





**Supplementary Figure 3.** **The within-cluster variation of conductivity (*W*_p_) for the average of random clustering, the hierarchical clustering (*C_1_*) and the spectral clustering (*C_2_*).** The values were normalized by the average *W*_p_ of random clustering. The random clustering was performed 100 times by dividing samples randomly into a given number of groups. The error bar shows the standard deviation from the 100 sampling. For the random clustering the *n_G_*-independent high values of calculated *W*_p_ showed the as-expected lack of power to separate Li-conductive and non-conductive phases. For ***C_1_*** and ***C_2_***, a large decrease of *W*_p_ occurs at *n*_G_=7, demonstrating the ***C***-σ_RT_ correlation at this critical number of groups. The calculated *W*_p_ is smaller for ***C_1_*** than that for ***C_2_***, which was attributed to the inclusion of β-Li_3_PS_4_ in the group containing Argyrodite, LLTO and LISICON in ***C_1_***.





**Supplementary Figure 4. Influence of simulated measurement noise on the within-cluster variation of conductivity (*W*_p_).** The values were normalized by the average *W*_p_ of random clusterings without simulated noise. The noise is simulated as a sample-independent Gaussian distribution $\varepsilon\sim\Delta\cdot N(0,1)$, where Δ is the strength of noise. The simulation was performed 100 times. The error bar shows the standard deviation from the 100 sampling. While the *W*_p_ increases with Δ, the observed decrease of *W*_p_ at the critical *n*_G_=7 is not affected, demonstrating the robustness of ***C_1_***- *σ*_RT_ correlation against the sample-independent experimental uncertainties.


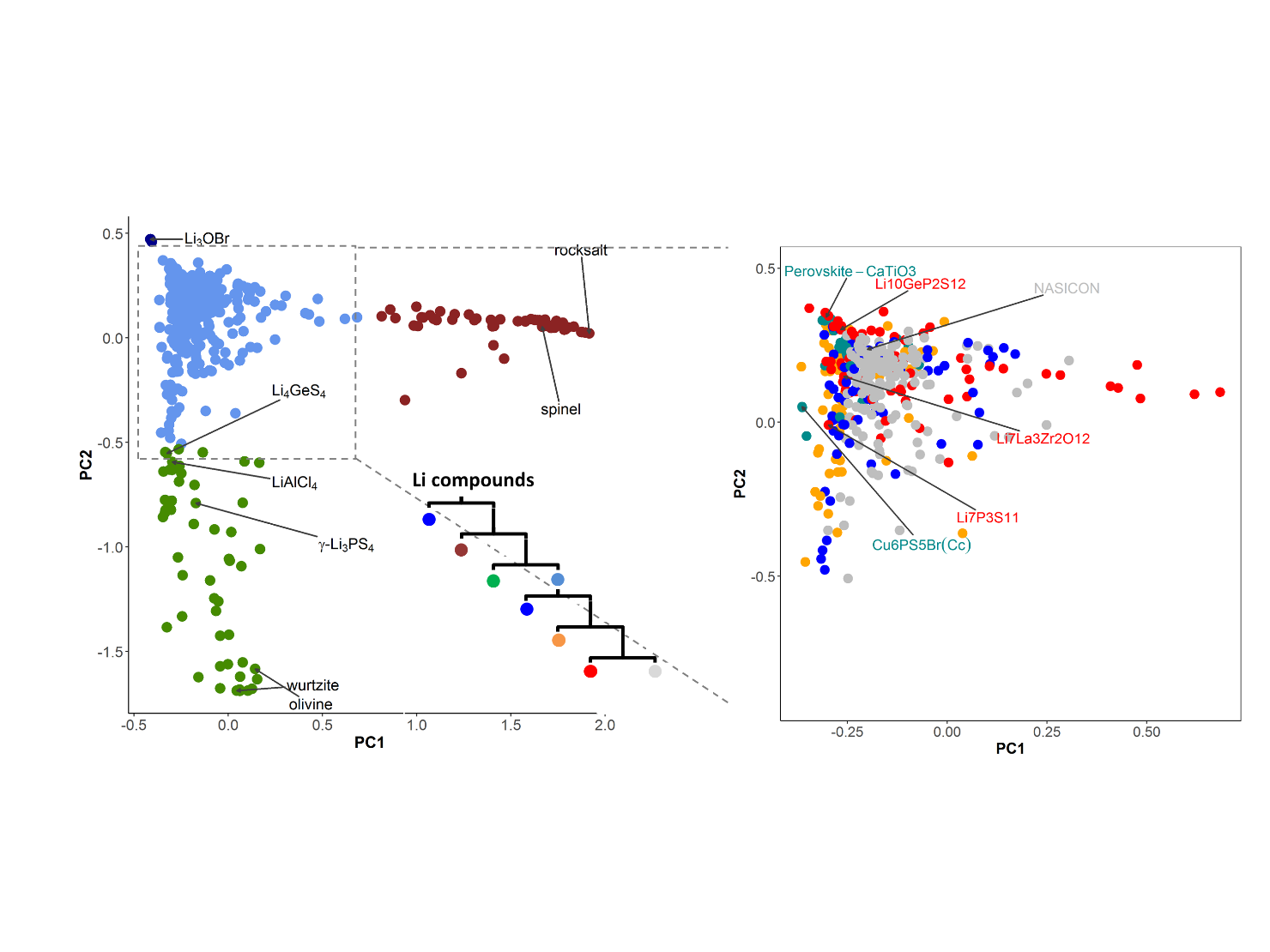


**Supplementary Figure 5. Results of C_2_ from the spectral clustering.** The samples are projected on the plane of the first and second principle component directions and colored based on the clustering results. The percentage in parentheses gives the proportion of variance on that principal axis. The insertion on the left shows the hierarchical structure of the clustering. The results show the clustering of known Li-ion conductors (right side), which is highly comparable with the ***C_1_*** results.





**Supplementary Figure 6. Optimizing the subset of mXRD representation using the coordinate descent algorithm.** In the original mXRD dataset, 32 dimensions were composed of all zeroes. The optimization is based on the reduction of the diffraction at special angels to minimize the within-cluster-variation of *σ*_RT_ (*W*_p_ function). After 8 iterations the dimensionality was reduced to 443 dimensions and the result stabilized afterwards.

**
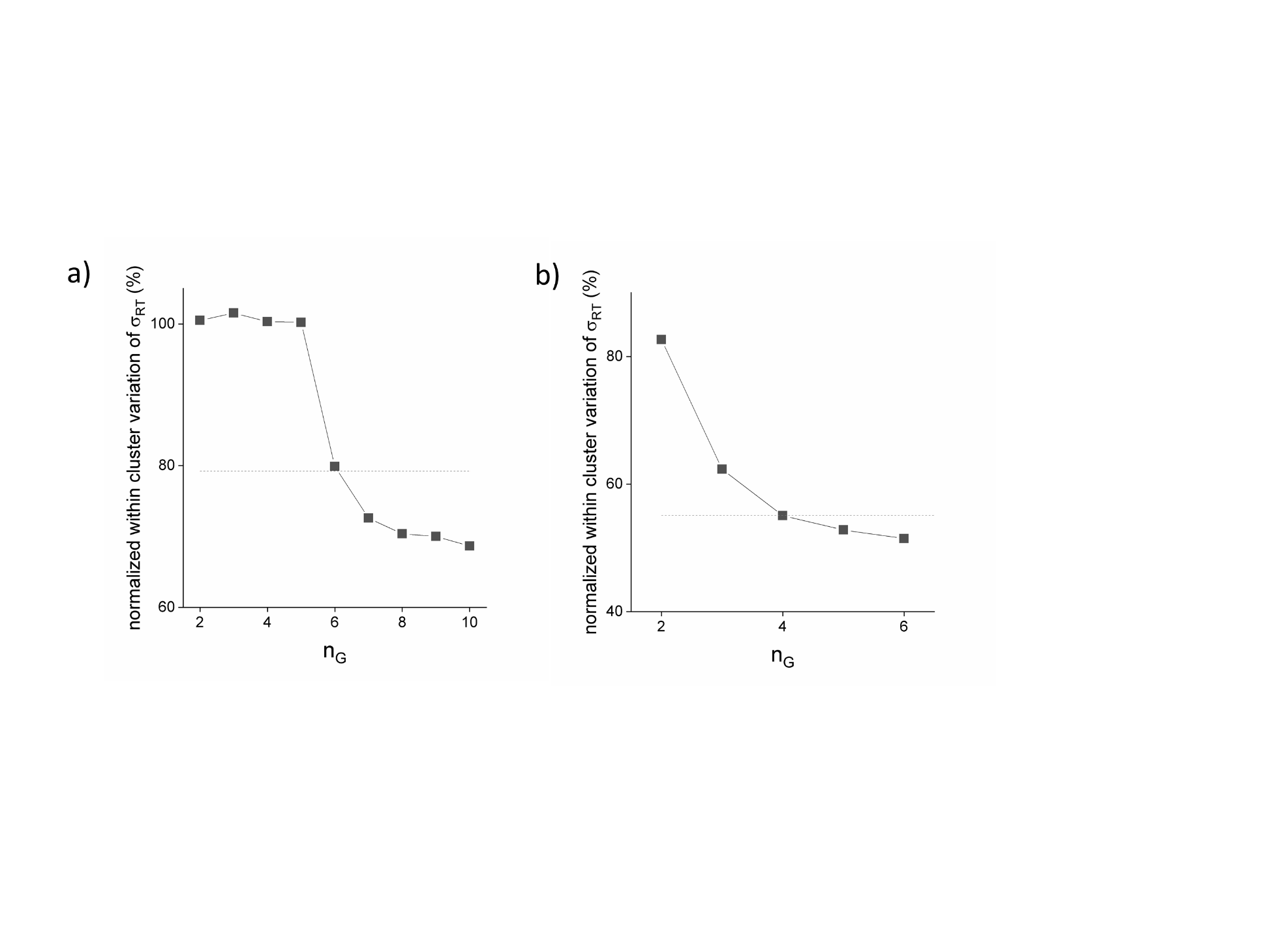
**

**Supplementary Figure 7. The within-cluster variation of conductivity (*W*_p_) for the clustering of all samples (a) and group IV to VII samples (b) for the clustering based on the optimized mXRD subset.** The values were normalized by the average *W*_p_ of random clustering. The dashed line shows the same score function calculated using the result of ***C_1_***. For the clustering of all samples, the largest drop of *W*_p_ corresponds to the clustering into eight groups while for the clustering of group IV to VIII samples the largest drop of *W*_p_ corresponds to the clustering into five groups. Compared to the clustering using the original mXRD data, the optimization of the mXRD subset reduced the *W*_p_ function in both cases.





**Supplementary Figure 8. Results of the *C_3_* based on the hierarchical clustering of the optimized subset of mXRD representation.** The clustering is projected on the plane of the first and second principle component directions of the optimized subset. The percentage in parentheses gives the proportion of variance on that principal axis. Note the principle components are for the mXRD subset and should not be confused with those for the original mXRD dataset. The clustering of known Li-ion conductors (solid symbols) are observed in three groups (red, yellow and blue).





**Supplementary Figure 9. Visualizing the samples in the groups having the congregation of known solid-state Li-ion conductors.** The samples are projected onto the plane spanned by the first and second principal axes of the full set of mXRD data (PC1 and PC2). The percentage in parentheses gives the proportion of variance on that principal axis. The color code of the labeled samples is kept consistent with those of symbols. Comparing to the results of *C_1_*, the only observable difference is the separation of β-Li_3_PS_4_ from the group containing Argyrodite, LLTO and LISICON in *C_2_*, and the clustering of LATP and LLTO in *C_3_*. Many non-overlapping samples appear at the boundaries, reflecting the influence of learning method on the clustering results.





**Supplementary Figure 10. Average mXRD spectrum for each group in *C_1_* clustering.** The shadow denotes the standard deviation of intensities at a given angle.


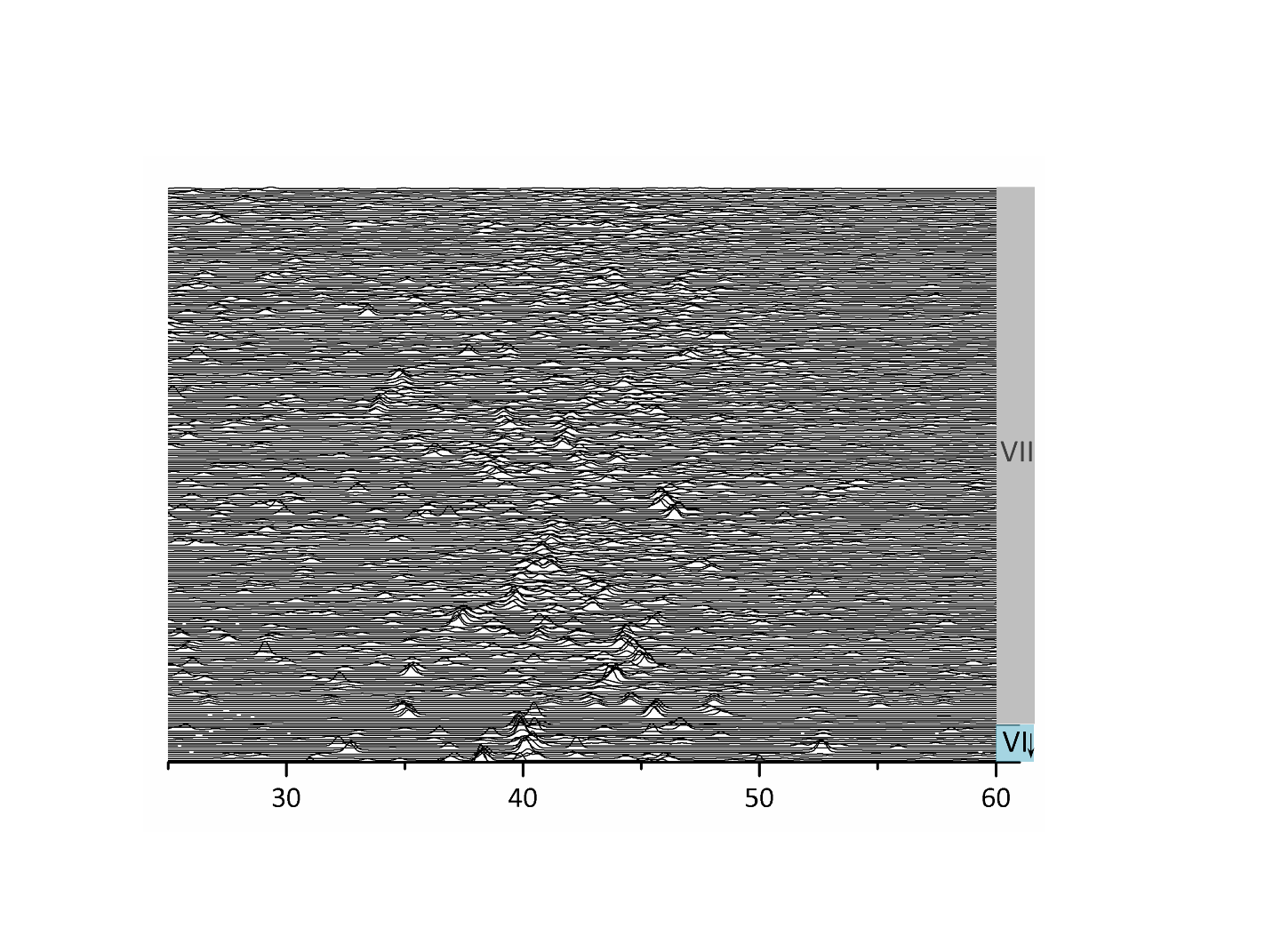


**Supplementary Figure 11. mXRD spectra of group VII samples.** The spectra look noise like and does not exhibit clear peaks, reflecting the highly distorted anion lattice in the group VII compounds.


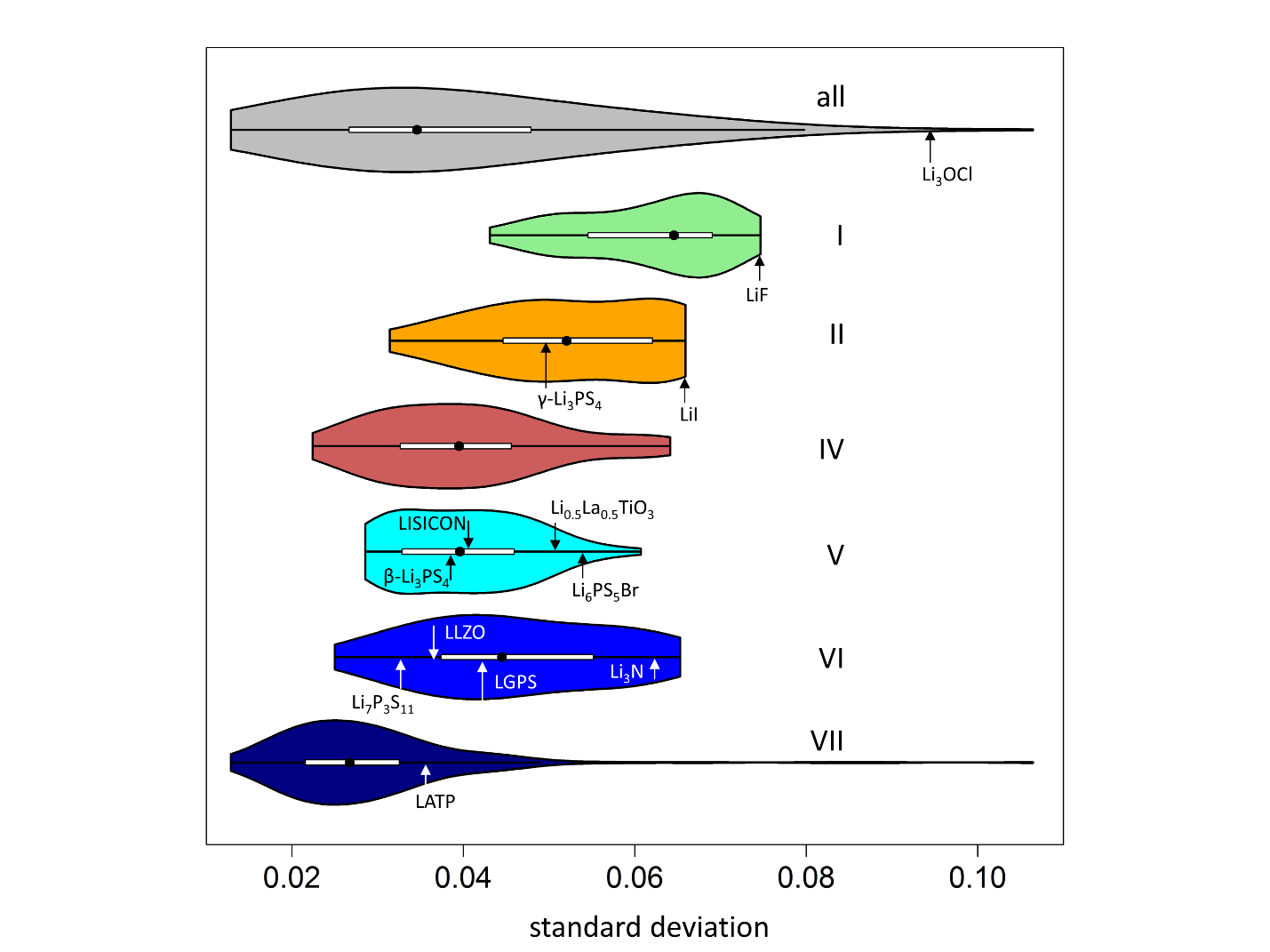


**Supplementary Figure 12. Violin plot of the standard deviation (SD) of mXRD for each group in *C_1_* classification.** From group I to VII in ***C_1_***, a shift of SD toward lower values was clearly observed. The SDs of all known SSLCs lie between 0.032 and 0.062.

**
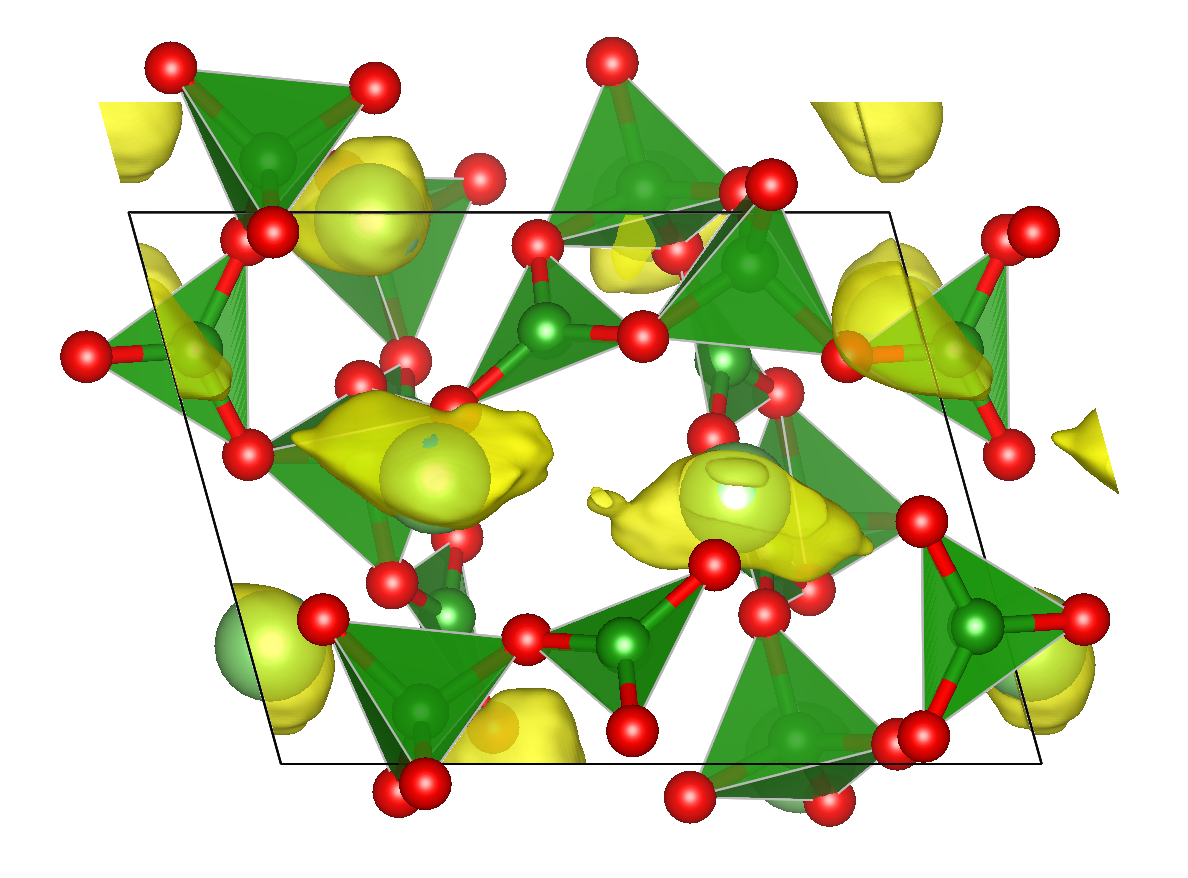
**

**Supplementary Figure 13.** Isosurfaces (yellow) of Li probability density for Li_3_B_7_O_12_ from Group VII from AIMD simulations. Legend: Li (light green), B (dark green), O (red). The local distortion of anion framework gives locally distorted positional distribution of Li ion but the non-optimal diffusion network impedes Li diffusion as shown by isolated isosurfaces of Li ions.


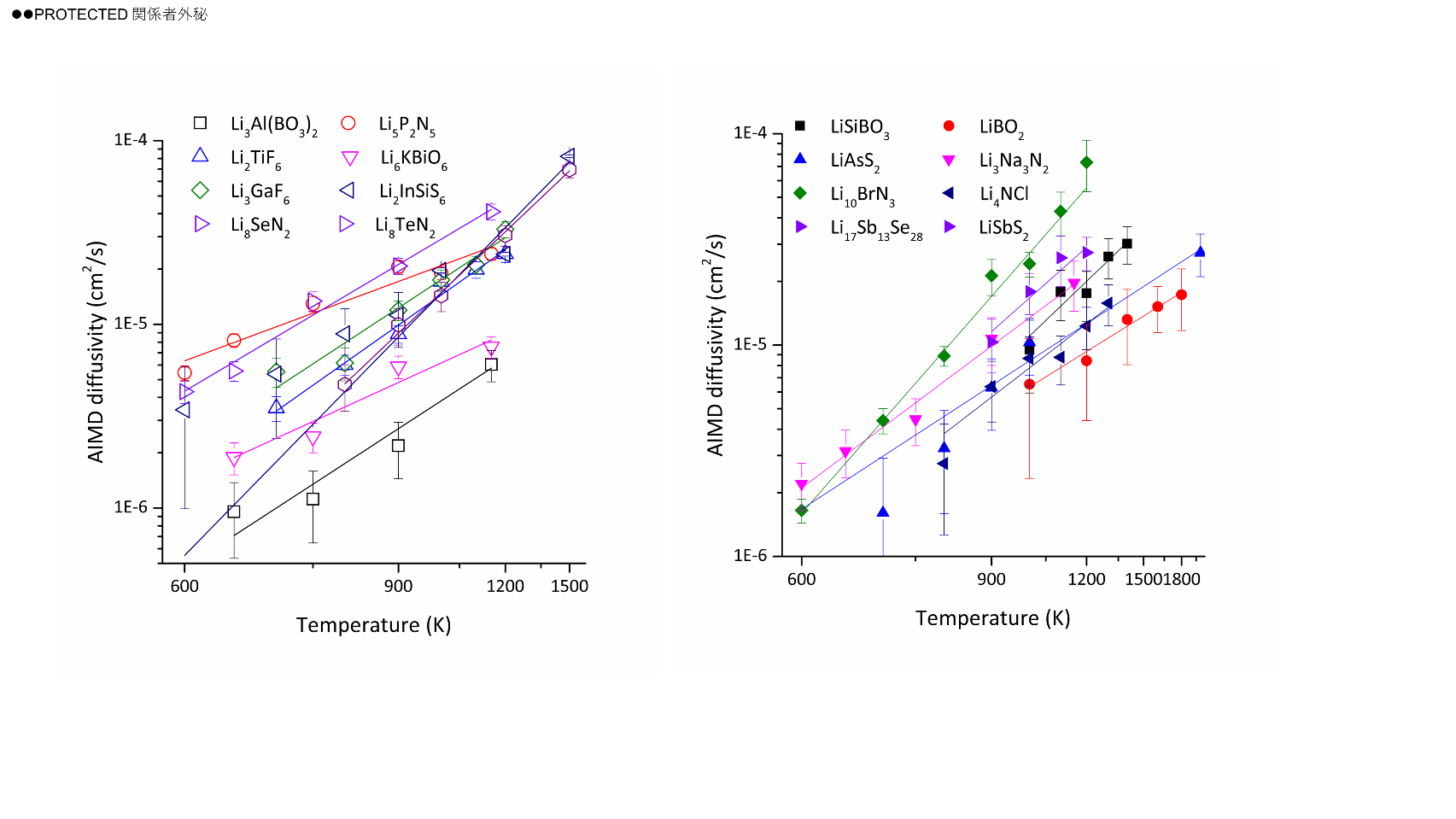


**Supplementary Figure 14. Diffusivity predicted from ab initio molecular dynamics simulations for compounds in Supplementary Table 2.** The simulations are typically performed at four to six different temperatures from 600 to 1200 K. The diffusivity of Li ions. The error bar shows the standard deviation of the conductivity estimated by the method in Ref. 63.

**
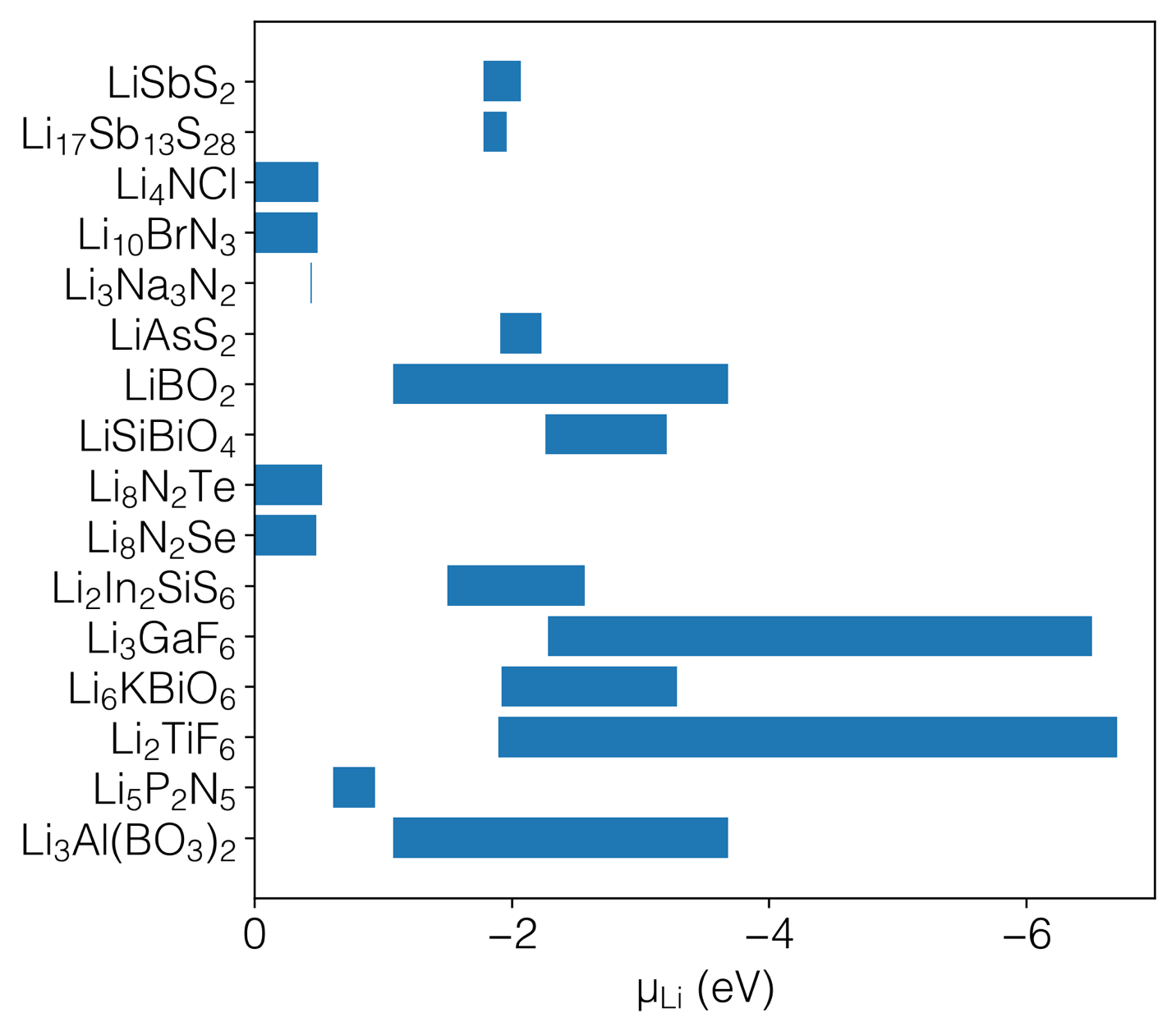
**

**Supplementary Figure 15. Thermodynamic intrinsic electrochemical window for compounds in Supplementary Table 2.** The calculations are performed using the data from the Materials Project using the scheme in Ref. 64.

**Supplementary Reference:**

1 Vijaymeena, M. K. & Kavitha, K. A Survey on Similarity Measures in Text Mining. *Machine Learning Appl. Int. J.* **3**, 19-28 (2016).

2 Yamane, H., Kikkawa, S. & Koizumi, M. High- and low-temperature phases of lithium boron nitride, Li3BN2: Preparation, phase relation, crystal structure, and ionic conductivity. *J. Solid State Chem.* **71**, 1-11 (1987).

3 Liu, Z. *et al.* Anomalous High Ionic Conductivity of Nanoporous β-Li3PS4. *J. Am. Chem. Soc.* **135**, 975-978 (2013).

4 Boulineau, S., Courty, M., Tarascon, J.-M. & Viallet, V. Mechanochemical synthesis of Li-argyrodite Li6PS5X (X = Cl, Br, I) as sulfur-based solid electrolytes for all solid state batteries application. *Solid State Ionics* **221**, 1-5 (2012).

5 Aono, H., Sugimoto, E., Sadaoka, Y., Imanaka, N. & Adachi, G.-y. Ionic Conductivity of Solid Electrolytes Based on Lithium Titanium Phosphate. *J. Electrochem. Soc.* **137**, 1023-1027 (1990).

6 Ibarra, J. *et al.* Influence of composition on the structure and conductivity of the fast ionic conductors La2/3−xLi3xTiO3 (0.03≤x≤0.167). *Solid State Ionics* **134**, 219-228 (2000).

7 Kamaya, N. *et al.* A lithium superionic conductor. *Nat. Mater.* **10**, 682-686 (2011).

8 Abrahams, I., Bruce, P. G., West, A. R. & David, W. I. F. Structure determination of LISICON solid solutions by powder neutron diffraction. *J. Solid State Chem.* **75**, 390-396 (1988).

9 Takada, K. *et al.* Lithium ion conduction in lithium magnesium thio-phosphate. *Solid State Ionics* **147**, 23-27 (2002).

10 Hasegawa, T. & Yamane, H. Synthesis and crystal structure analysis of Li2NaBP2O8 and LiNa2B5P2O14. *J. Solid State Chem.* **225**, 65-71 (2015).

11 Kim, C.-S., Hwang, Y.-H., Kim, H. K. & Kim, J. N. Ionic conductivity of crystalline and glassy Li2B4O7 *Phys. Chem. Glasses* **44**, 166-169 (2003).

12 Kanno, R. *et al.* Ionic Conductivity and Phase Transition of the Bromide Spinels, Li2 − 2x  M 1 + x Br4 (  M  = Mg , Mn )  *J. Electrochem. Soc.* **133**, 1052-1056 (1986).

13 Narimatsu, E., Yamamoto, Y., Takeda, T., Nishimura, T. & Hirosaki, N. High lithium conductivity in Li1-2xCaxSi2N3. *J. Mater. Res.* **26**, 1133-1142 (2011).

14 Brant, J. A. *et al.* Fast Lithium Ion Conduction in Li2SnS3: Synthesis, Physicochemical Characterization, and Electronic Structure. *Chem. Mater.* **27**, 189-196 (2015).

15 Rao, S. R., Lingam, C. B., Rajesh, D., Vijayalakshmi, R. P. & Sunandana, C. S. Structural, conductivity and dielectric properties of Li2SO4. *Eur. Phys. J. -Appl. Phys.* **66**, 30906 (2014).

16 Ohsuka, H. & Yamaji, A. Preparation and electrical conductivity of LISICON thin films. *Solid State Ionics* **8**, 43-48 (1983).

17 Maekawa, H., Iwatani, T., Shen, H., Yamamura, T. & Kawamura, J. Enhanced lithium ion conduction and the size effect on interfacial phase in Li2ZnI4–mesoporous alumina composite electrolyte. *Solid State Ionics* **178**, 1637-1641 (2008).

18 Hellstrom, E. E. & Gool, W. V. Li ion conduction in Li2ZrO3, Li4ZrO4, and LiScO2. *Solid State Ionics* **2**, 59-64 (1981).

19 Yamane, H., Kikkawa, S. & Koizumi, M. Lithium aluminum nitride, Li3AlN2 as a lithium solid electrolyte. *Solid State Ionics* **15**, 51-54 (1985).

20 Subban, C. V. *et al.* Search for Li-electrochemical activity and Li-ion conductivity among lithium bismuth oxides. *Solid State Ionics* **283**, 68-74 (2015).

21 Lapp, T., Skaarup, S. & Hooper, A. Ionic conductivity of pure and doped Li3N. *Solid State Ionics* **11**, 97-185 (1983).

22 Huang, F. Q., Yao, J., Liu, Z., Yang, J. & Ibers, J. A. Synthesis, structure, and ionic conductivity of Na5Li3Ti2S8. *J. Solid State Chem.* **181**, 837-841 (2008).

23 Zhao, Y. & Daemen, L. L. Superionic Conductivity in Lithium-Rich Anti-Perovskites. *J. Am. Chem. Soc.* **134**, 15042-15047 (2012).

24 Ivonov-Shitz, A. K., Kireev, V. V., Mel'nikov, O. K. & Demianets, L. N. Growth and ionic conductivity of γ-Li3PO4. *Crstallog. Rep.* **46**, 864-867 (2001).

25 Olivier-Fourcade, J., Maurin, M. & Philippot, E. Modification de la nature de la conductivite electrique par creation de sites vacants dans les phases a caractere semi-conducteur du systeme Li2SSb2S3. *Solid State Ionics* **9-10**, 135-137 (1983).

26 Hodge, I. M., Ingram, M. D. & West, A. R. Ionic Conductivity of Li4SiO4, Li4GeO4, and Their Solid Solutions. *J. Am. Ceram. Soc.* **59**, 360-366 (1976).

27 Murayama, M., Sonoyama, N., Yamada, A. & Kanno, R. Material design of new lithium ionic conductor, thio-LISICON, in the Li2S–P2S5 system. *Solid State Ionics* **170**, 173-180 (2004).

28 Hood, Z. D. *et al.* Structural and electrolyte properties of Li4P2S6. *Solid State Ionics* **284**, 61-70 (2016).

29 Khorassani, A. & West, A. R. Li+ ion conductivity in the system Li4SiO4—Li3VO4. *J. Solid State Chem.* **53**, 369-375 (1983).

30 Ahn, B. T. & Huggins, R. A. Synthesis and lithium conductivities of Li2SiS3 and Li4SiS4. *Mater. Res. Bull.* **24**, 889-897 (1989).

31 Cordier, G., Gudat, A. & Rabenau, A. LiCaN and Li4SrN2, Derivatives of the Fluorite and Lithium Nitride Structures. *Angew. Chem. Int. Ed.* **28**, 1702-1703 (1989).

32 Raistrick, I. D., Ho, C. & Huggins, R. A. Lithium ion conduction in Li5AlO4, Li5GaO4 and Li6ZnO4. *Mater. Res. Bull.* **11**, 953-957 (1976).

33 Murugan, R., Weppner, W., Schmid-Beurmann, P. & Thangadurai, V. Structure and lithium ion conductivity of bismuth containing lithium garnets Li5La3Bi2O12 and Li6SrLa2Bi2O12. *Mater. Sci. Eng. B* **143**, 14-20 (2007).

34 Hartwig, P., Weppner, W. & Wichelhaus, W. Fast ionic lithium conduction in solid lithium nitride chloride. *Mater. Res. Bull.* **14**, 493-498 (1979).

35 Deng, Y. *et al.* Structural and Mechanistic Insights into Fast Lithium-Ion Conduction in Li4SiO4-Li3PO4 Solid Electrolytes. *J. Am. Chem. Soc.* **137**, 9136-9145 (2015).

36 Burmakin, E. I., Shehtman, G. S. & Alikin, V. N. Ionic Conductivity of Li6Ge2O7 and its Solid Solutions *Mater. Sci. Forum* **76**, 107-110 (1991).

37 Howard, M. A., Clemens, O., Slater, P. R. & Anderson, P. A. Hydrogen absorption and lithium ion conductivity in Li6NBr3. *J. Alloy Comp.* **645**, S178-S183 (2015).

38 Obayashi, H., Gotoh, A. & Nagai, R. Composition dependence of lithium ionic conductivity in lithium nitride-lithium iodide system. *Mater. Res. Bull.* **16**, 581-585 (1981).

39 Pantyukhina, M. I., Shchelkanova, M. S., Stepanov, A. P. & Buzlukov, A. L. Investigation of ion transport in Li8ZrO6 and Li6Zr2O7 solid electrolytes. *Bull. Rus. Acad. Sci. Phys.* **74**, 653-655 (2010).

40 Muhle, C., Dinnebier, R. E., Wullen, L. v., Schwering, G. & Jansen, M. New insights into the structural and dynamical features of lithium hexaoxometalates Li7MO6 (M = Nb, Ta, Sb, Bi). *Inorg. Chem.* **43**, 874-881 (2004).

41 Mizuno, F., Hayashi, A., Tadanaga, K. & Tatsumisago, M. New, Highly Ion‐Conductive Crystals Precipitated from Li2S–P2S5 Glasses. *Adv. Mater.* **17**, 918-921 (2005).

42 Schnick, W. & Luecke, J. Lithium ion conductivity of LiPN2 and Li7PN4. *Solid State Ionics*, 271 (1990).

43 Miara, L. J. *et al.* Li-ion conductivity in Li9S3N *J. Mater. Chem. A* **3**, 20338-20344 (2015).

44 Weppner, W. & Huggins, R. A. Ionic Conductivity of Solid and Liquid LiAlCl4. *J. Electrochem. Soc.* **124**, 35-38 (1977).

45 Miyazaki, R. & Maekawa, H. Li+-Ion Conduction of Li3AlF6 Mechanically Milled with LiCl *ECS Electrochem. Lett.* **1**, A87-A89 (2012).

46 Ross, S., Welsch, A.-M. & Behrens, H. Lithium conductivity in glasses of the Li2O–Al2O3–SiO2 system *Phys. Chem. Chem. Phys.* **17**, 465-474 (2015).

47 Johnson, R. T., Morosin, B., Knotek, M. L. & Biefeld, R. M. Ionic conductivity in LiAlSiO4. *Phys. Lett. a* **54**, 403-404 (1975).

48 Cakmak, G., Nuss, J. & Jansen, M. LiB6O9F, the First Lithium Fluorooxoborate – Crystal Structure and Ionic Conductivity. *Z. Anorg. Allg. Chem.* **635**, 631-636 (2009).

49 Krichen, M., Megdiche, M., Guidara, K. & Gargouri, M. AC conductivity and mechanism of conduction study of lithium barium pyrophosphate Li2BaP2O7 using impedance spectroscopy *Ionics* **21**, 935-948 (2015).

50 Heitjans, P., Tobschall, E. & Wilkening, M. Ion transport and diffusion in nanocrystalline and glassy ceramics *Eur. Phys. J.* **161**, 97-108 (2008).

51 Kanno, R., Takeda, Y. & Yamamoto, O. Ionic conductivity of solid lithium ion conductors with the spinel structure: Li2MCl4 (M = Mg, Mn, Fe, Cd). *Mater. Res. Bull.* **16**, 999-1005 (1981).

52 Seo, I. & Kim, Y. Synthesis and characterization of lithium germanogallium sulfide, Li2GeGa2S6. *Solid State Ionics* **261**, 106-110 (2014).

53 Almond, D. P., Hunter, C. C. & West, A. R. The extraction of ionic conductivities and hopping rates from a.c. conductivity data. *J. Mater. Sci.* **19**, 3236-3248 (1984).

54 Quintana, P. & West, A. R. Conductivity of lithium gallium silicates. *Solid State Ionics* **23**, 179-187 (1987).

55 Mugnier, Y., Galez, C., Crettez, J. M., Bourson, P. & Bouillot, J. Dielectric characterization and ionic conductivity of α-LiIO3 crystals related to the growth conditions. *Solid State Commun.* **115**, 619-623 (2000).

56 Salanne, M., Marrocchelli, D. & Watson, G. A Cooperative Mechanism for the Diffusion of Li+ Ions in LiMgSO4F. *J. Phys. Chem. C* **116**, 18618-18625 (2012).

57 Mellander, B.-E., Graneli, B. & Roos, J. Ionic conductivity of single crystal LiNaSO4. *Solid State Ionics* **40-41**, 162-164 (1990).

58 Hockicko, P., Kudelcik, J., Munoz, F. & Munoz-Senovilla, L. Structural and Electrical Properties of LiPO3 Glasses. *Appl. Phys.* **2**, 198-205 (2015).

59 Yamane, H., Kikkawa, S. & Koizumi, M. Preparation of lithium silicon nitrides and their lithium ion conductivity. **25**, 183-191 (1987).

60 Barpanda, P. *et al.* iZnSO4F Made in an Ionic Liquid: A Ceramic Electrolyte Composite for Solid‐State Lithium Batteries. *Angew. Chem. Int. Ed.* **50**, 2526-2531 (2011).

61 Shimonishi, Y. *et al.* Synthesis of garnet-type Li7− xLa3Zr2O12− 1/2x and its stability in aqueous solutions. *Solid State Ionics* **183**, 48-53 (2011).

62 Kokal, I., Somer, M., Notten, P. H. L. & Hintzen, H. T. Sol–gel synthesis and lithium ion conductivity of Li7La3Zr2O12 with garnet-related type structure. *Solid State Ionics* **185**, 42-46 (2011).

63 He, X., Zhu, Y., Epstein, A. & Mo, Y. Statistical Variances of Diffusional Properties from Ab Initio Molecular Dynamics Simulations. *Npj Comp. Mater.* **4**, 18 (2018).

64 Zhu, Y., He, X. & Mo, Y. Origin of Outstanding Stability in the Lithium Solid Electrolyte Materials: Insights from Thermodynamic Analyses Based on First-Principles Calculations. *ACS Appl. Mater. Interfaces* **7**, 23685-23693 (2015).
